# Associations of traditional cardiovascular risk factors with 15-year blood pressure change and trajectories in Chinese adults: a prospective cohort study

**DOI:** 10.1097/HJH.0000000000003717

**Published:** 2024-03-22

**Authors:** Yiqian Zhang, Qiufen Sun, Canqing Yu, Dianjianyi Sun, Yuanjie Pang, Pei Pei, Huaidong Du, Ling Yang, Yiping Chen, Xiaoming Yang, Xiaofang Chen, Junshi Chen, Zhengming Chen, Liming Li, Jun Lv

**Affiliations:** a Department of Epidemiology & Biostatistics, School of Public Health, Peking University; b Peking University Center for Public Health and Epidemic Preparedness & Response, Beijing, China; c Key Laboratory of Epidemiology of Major Diseases (Peking University), Ministry of Education; d Medical Research Council Population Health Research Unit at the University of Oxford; e Clinical Trial Service Unit & Epidemiological Studies Unit (CTSU), Nuffield Department of Population Health, University of Oxford, Oxford, United Kingdom; f Chengdu Medical College, Chengdu, Sichuan, China; g China National Center for Food Safety Risk Assessment, Beijing, China; h State Key Laboratory of Vascular Homeostasis and Remodeling, Peking University

**Keywords:** blood pressure change, cumulative blood pressure, cardiovascular disease risk factors, trajectory

## Abstract

**Objective:**

How traditional cardiovascular disease (CVD) risk factors are related to long-term blood pressure change (BPC) or trajectories remain unclear. We aimed to examine the independent associations of these factors with 15-year BPC and trajectories in Chinese adults.

**Methods:**

We included 15 985 participants who had attended three surveys, including 2004–2008 baseline survey, and 2013–2014 and 2020–2021 resurveys, over 15 years in the China Kadoorie Biobank (CKB). We measured systolic and diastolic blood pressure (SBP and DBP), height, weight, and waist circumference (WC). We asked about the sociodemographic characteristics and lifestyle factors, including smoking, alcohol drinking, intake of fresh vegetables, fruits, and red meat, and physical activity, using a structured questionnaire. We calculated standard deviation (SD), cumulative blood pressure (cumBP), coefficient of variation (CV), and average real variability (ARV) as long-term BPC proxies. We identified blood pressure trajectories using the latent class growth model.

**Results:**

Most baseline sociodemographic and lifestyle characteristics were associated with cumBP. After adjusting for other characteristics, the cumSBP (mmHg × year) increased by 116.9 [95% confidence interval (CI): 111.0, 122.7] for every 10 years of age. The differences of cumSBP in heavy drinkers of ≥60 g pure alcohol per day and former drinkers were 86.7 (60.7, 112.6) and 48.9 (23.1, 74.8) compared with less than weekly drinkers. The cumSBP in participants who ate red meat less than weekly was 29.4 (12.0, 46.8) higher than those who ate red meat daily. The corresponding differences of cumSBP were 127.8 (120.7, 134.9) and 70.2 (65.0, 75.3) for BMI per 5 kg/m^2^ and WC per 10 cm. Most of the findings of other BPC measures by baseline characteristics were similar to the cumBP, but the differences between groups were somewhat weaker. Alcohol drinking was associated with several high-risk trajectories of SBP and DBP. Both BMI and WC were independently associated with all high-risk blood pressure trajectories.

**Conclusions:**

Several traditional CVD risk factors were associated with unfavorable long-term BPC or blood pressure trajectories in Chinese adults.

## INTRODUCTION

Hypertension remains a major public health problem worldwide and is a modifiable risk factor for noncommunicable diseases such as cardiovascular disease (CVD), chronic kidney disease, and dementia. The Global Burden of Disease study indicated that high systolic blood pressure (SBP) was the leading cause of the global burden of disease, being responsible for 19.2% of all deaths globally and 9.3% of attributable disability-adjusted life-years (DALYs) in 2019 [1].

Most previous epidemiological studies of blood pressure have focused on a single measurement at one-time point or blood pressure change between two-time points. However, a few studies suggest that long-term multiple recordings of blood pressure may have greater value in predicting disease development and prognosis [2]. Some indices, including the standard deviation (SD), coefficient of variation (CV), average real variability (ARV), and cumulative blood pressure (cumBP), have been used to reflect the long-term blood pressure change (BPC). Several population-based prospective studies demonstrated that every 1-SD rise in SD (≈5–10 mmHg), CV (≈5%), and ARV (≈3.6 mmHg) of SBP increased the risk for all-cause mortality or CVD incidence by 14–21%, 5–13%, and 15–26%, respectively [3–7]. The cumBP is a measure that integrates long-term severity and duration of blood pressure exposure. The risk of CVD incidence for each 1-SD increase in cumSBP and diastolic blood pressure (DBP) (≈101 /82 mmHg × year) raised 55% and 50%, respectively [6]. Blood pressure trajectory, taking both the average blood pressure levels and the blood pressure changes over time into consideration, also has additional value for predicting CVD risk [8].

There are quite limited studies on the independent association between traditional CVD risk factors and long-term BPC or trajectories. It remains unclear whether the risk factors for unfavorable long-term blood pressure fluctuation are the same as those for a single or short-term blood pressure level. Therefore, we aimed to examine the independent associations of traditional CVD risk factors, including sociodemographic characteristics and lifestyle factors, with long-term BPC and trajectories in the China Kadoorie Biobank (CKB) of more than 15 000 participants who attended three-round surveys within fifteen years. This study will help understand which factors may increase CVD risk through unfavorable long-term BPC or trajectories.

## METHODS

### Study population

The CKB baseline survey was done between 2004 and 2008 in five urban and five rural areas across China, including more than 0.5 million participants aged 30–79. Details of the study design have been described previously [9]. About 5% of surviving participants were cluster sampled for periodic resurveys every 4–5 years. The priority was given to those who participated in previous resurveys, supplemented by some new cluster samples from those recruited at baseline. The present study used data from the baseline survey and resurveys of 2013–2014 (*n* = 25 069) and 2020–2021 (*n* = 23 753). The CKB was approved by the Ethics Review Committee of the Chinese Center for Disease Control and Prevention (Beijing, China) and the Oxford Tropical Research Ethics Committee, University of Oxford (Oxford, UK). All participants provided written informed consent.

In the present analysis, we included 15 986 participants who attended two resurveys and had blood pressure measurements available across three visits. We excluded participants with missing data for body mass index (BMI) at 2004–2008 baseline (*n* = 1), leaving 15 985 participants for the final analysis.

### Assessment of blood pressure and other variables

Blood pressure measurement was conducted after the on-site registration and physical measurements, including height, weight, and waist circumference (WC), but before the lung function test, blood collection, questionnaire, and other survey items. Blood pressure was measured using UA-779 digital sphygmomanometer for 2004–2008 baseline survey, Omron HEM-7430 sphygmomanometer for 2013–2014 resurvey, and UA-767PBT-Ci digital blood pressure monitor for 2020–2021 resurvey. The sphygmomanometers were uniformly calibrated, and the blood pressure was measured using the same standard operating procedures at each visit. After participants had sat for at least 5 min, trained investigators measured blood pressure twice from the right upper arm, with at least a 10-s cuff released interval between measurements. If the difference between the two SBP readings was more than 10 mmHg, a third measurement was taken after a 1-min rest. The last two readings were recorded, and their average was used for analyses [10].

The sociodemographic characteristics (e.g., age, sex, and level of education), lifestyle factors (e.g., smoking, alcohol consumption, habitual dietary intake, and physical activity), and medication status were collected using interviewer-administered questionnaires. All data were directly entered into a laptop-based system with built-in functions to avoid missing and logical errors in all three visits. The assessment and definition of lifestyle factors have been described elsewhere [11–15] and are also presented in the supplementary methods. Height, weight, and WC were measured by trained staff using uniformly equipped instruments. BMI was calculated as weight divided by the square of height (kg/m^2^).

Participants who met any of the following conditions were defined as having hypertension: self-reports of being diagnosed with hypertension by hospitals at township/district level or above, receiving antihypertensive treatment, had mean SBP ≥140 mmHg or mean DBP ≥90 mmHg recorded in two blood pressure measurements.

### Statistical analysis

According to previous studies, we added 15 mmHg to the measured SBP and 10 mmHg to the measured DBP in antihypertensive-treated participants [16]. The mean SBP and DBP were calculated across three visits for each participant. We calculated the SD and cumBP as primary measures of long-term BPC. The SD of blood pressure was calculated as:


$$\text{SD}\;=\;\sqrt{\frac{\sum_{i=1}^{n}(\text{B}{\text{P}}_{n}-\bar{\text{BP}}{)}^{2}}{n-1}}.$$

The cumBP was the area under the curve of long-term blood pressure and was calculated using the following formula [17]:


$$\text{cumBP}\;=\;\left(\frac{\text{B}{\text{P}}_{1}+\text{B}{\text{P}}_{2}}{2}\;\times T_{12}\right)\;+\;\left(\frac{\text{B}{\text{P}}_{2}+\text{B}{\text{P}}_{3}}{2}\;\times \;T_{23}\right),$$

where BP_*n*_ is the blood pressure at visit *n*, $\bar{\text{BP}}$ is the mean blood pressure, and *T*_*ab*_ is the number of years between visits *a* and *b*. We also included the CV and ARV as supplementary analyses (supplementary methods), considering these two indices have strong correlations with SD [18]. We computed Spearman's rank correlation coefficients between index metrics to understand their interrelationships.

We used latent class growth models to identify participants with similar underlying trajectories of change in SBP and DBP over a 15-year period (detailed in the supplementary methods). The best-fit model was selected based on the minimum Bayesian information criterion (BIC), an average posterior probability of more than 0.7 for each trajectory group, and membership in each trajectory group of more than 2%. Trajectories with a mean blood pressure level lower than the 140/90 mmHg threshold of hypertension were accounted as low-risk, otherwise, high-risk. We further regrouped SBP and DBP trajectories into four combined groups: both SBP and DBP low-risk trajectories, isolated SBP high-risk trajectory (high-risk SBP trajectory but low-risk DBP trajectory), isolated DBP high-risk trajectory (high-risk DBP trajectory but low-risk SBP trajectory), and both SBP and DBP high-risk trajectories.

We examined hypertension prevalence, differences in long-term BPC measures, and compositions of SBP, DBP, and combined blood pressure trajectories by baseline sociodemographic and lifestyle factors. The general linear regression model was used to calculate the differences in long-term BPC measures by baseline characteristics, with adjustment for all other characteristics analyzed except for body shape, which was defined by BMI and WC. Specifically, the variables included in the model were age (year), sex (men and women), residence (10 study areas), education (no formal school, primary school, middle school, high school, technical school or college, and university), smoking (never, former, and current <20 or ≥20 cigarettes or equivalent per day), alcohol drinking (less than weekly, former, weekly, and <30, 30–59 or ≥60 g pure alcohol per day), intake frequency of fresh vegetable, fresh fruit, and red meat (days per week), total physical activity level (metabolic equivalent task-hour per day), BMI (kg/m^2^), and WC (cm). To avoid the multicollinearity between BMI and WC, we first regressed BMI and WC on each other. The residuals from this analysis were included in the model as covariates instead of BMI or WC [19]. BMI and WC were not included in the covariates for body shape analysis. The adjusted differences and 95% confidence interval (CI) in BPC measures compared with the reference group were presented for categorical variables. The adjusted BPC change and 95% CI per specified unit were presented for continuous variables. We also repeated the above analyses in participants with hypertension (those with hypertension at any of the three surveys), normotension (those who maintained normotension across three surveys), and without antihypertensive treatment across three surveys separately. The multinomial logistic regression model was used to calculate the adjusted relative risk ratio (RRR) and 95% CI of baseline characteristics with SBP trajectories, DBP trajectories, and combined blood pressure trajectories, with the same covariate adjustment as above.

The blood pressure trajectory modeling was performed using the *traj* Stata plugin [20], and all analyses were performed using Stata (version 15.0). The level of statistical significance was defined as two-sided *P* < 0.05.

## RESULTS

### Characteristics of the study population

A total of 15 985 participants were included in the analysis, of which 63.9% were women and 32.1% resided in urban areas. The average interval was 8.0 years between 2004 and 2008 baseline survey and 2013–2014 resurvey, and 7.3 years between 2013–2014 resurvey and 2020–2021 resurvey. The mean age of participants was 50.2 ± 9.2 years at 2004–2008 baseline, 58.2 ± 9.2 years at 2013–2014 resurvey, and 65.5 ± 9.2 years at 2020–2021 resurvey. Corresponding to the three surveys, the prevalence of hypertension was 33.3%, 48.6%, and 59.3%; and the percentage of participants with hypertension who received antihypertensive treatment was 28.4%, 52.5%, and 69.4% (Table S1, Supplemental Digital Content). As displayed in Table 1, there was a higher prevalence of hypertension among participants who were older, less educated, former smokers, former or current daily alcohol drinkers, and overweight or obese.

**TABLE 1:** Hypertension prevalence at baseline and resurveys by baseline characteristics of the study participants

			Hypertension prevalence (%)		
	*N* (%)	Average age at baseline (SD)	2004–2008 baseline	2013–2014 resurvey	2020–2021 resurvey
All participants	15 985	50.2 (9.2)	33.3	48.6	59.3
Age, years					
<50	7797 (48.8)	42.4 (4.5)	21.7	35.0	48.7
50–59	5720 (35.8)	54.6 (2.8)	40.1	57.0	66.8
≥60	2468 (15.4)	64.8 (3.6)	54.1	71.9	75.4
Sex					
Women	10 220 (63.9)	49.8 (9.1)	33.0	48.6	58.3
Men	5765 (36.1)	51.0 (9.2)	33.8	48.5	61.1
Residence					
Urban	5139 (32.1)	51.0 (8.9)	33.2	54.5	59.7
Rural	10 846 (67.9)	49.9 (9.3)	33.4	45.7	59.1
Education					
College or university	529 (3.3)	47.8 (9.9)	25.3	42.7	50.5
Middle or high school	6362 (39.8)	46.5 (8.1)	27.3	43.1	55.4
Primary school or below	9094 (56.9)	53.0 (8.8)	38.0	52.7	62.6
Smoking					
Never	11 393 (71.3)	49.8 (9.2)	33.4	48.9	58.6
Former	723 (4.5)	54.8 (9.0)	43.3	59.8	68.0
Current, cigarettes (or equivalent)/day					
< 20	1718 (10.7)	51.6 (9.2)	30.9	45.1	58.7
≥ 20	2151 (13.5)	50.0 (8.5)	31.5	45.8	60.6
Alcohol drinking					
Less than weekly	13 220 (82.7)	50.0 (9.2)	32.7	48.2	58.2
Former	536 (3.4)	54.5 (8.6)	40.3	54.9	68.5
Weekly	886 (5.5)	47.6 (8.7)	33.5	46.8	62.4
Daily, g/day (pure alcohol)					
< 30	327 (2.0)	52.9 (9.0)	35.5	49.5	62.1
30–59	433 (2.7)	52.4 (8.6)	33.7	51.7	63.3
≥ 60	583 (3.6)	51.6 (8.6)	38.3	51.1	66.9
Fresh vegetable consumption					
Daily	15 262 (95.5)	50.2 (9.1)	33.2	48.6	59.5
Nondaily	723 (4.5)	49.9 (9.3)	36.4	47.3	55.9
Fresh fruit consumption					
Daily	2190 (13.7)	50.2 (9.3)	32.0	48.8	56.9
Nondaily	13 795 (86.3)	50.2 (9.1)	33.5	48.5	59.7
Red meat consumption					
Daily	3917 (24.5)	49.8 (8.8)	31.4	49.1	57.8
Weekly	9277 (58.0)	50.4 (9.3)	33.0	48.4	59.0
Less than weekly	2791 (17.5)	50.1 (9.2)	36.9	48.4	62.5
Total physical activity^a^					
Low	5335 (33.4)	50.7 (9.2)	34.5	51.5	62.9
Medium	5330 (33.3)	50.2 (9.3)	32.2	47.8	57.4
High	5320 (33.3)	49.8 (8.9)	33.2	46.4	57.6
BMI					
<18.5 kg/m^2^	582 (3.6)	51.8 (10.5)	17.4	29.6	37.3
18.5–23.9 kg/m^2^	8521 (53.3)	50.0 (9.4)	25.7	40.5	51.6
24.0–27.9 kg/m^2^	5294 (33.1)	50.4 (8.7)	41.0	57.4	68.7
≥28.0 kg/m^2^	1588 (9.9)	50.5 (8.6)	54.4	69.4	77.2
WC (men/women)					
<85/80 cm	10 153 (63.5)	49.7 (9.4)	26.0	40.7	52.3
85–90/80–85 cm	2740 (17.1)	50.5 (8.7)	40.4	57.3	67.2
≥90/85 cm	3092 (19.3)	51.7 (8.6)	50.9	66.6	75.4
Body shape					
BMI <18.5	582 (3.6)	51.8 (10.5)	17.4	29.6	37.3
BMI 18.5–23.9, WC <90/85	8368 (52.3)	49.9 (9.4)	25.4	40.2	51.4
BMI 18.5–23.9, WC ≥90/85	153 (1.0)	54.8 (9.1)	44.4	58.2	64.7
BMI 24.0–27.9, WC <90/85	3726 (23.3)	49.6 (8.7)	38.4	54.6	66.4
BMI 24.0–27.9, WC ≥90/85	1568 (9.8)	52.3 (8.5)	47.1	64.0	74.2
BMI ≥28.0, WC <90/85	217 (1.4)	48.7 (8.7)	43.8	62.2	72.4
BMI ≥28.0, WC ≥90/85	1371 (8.6)	50.7 (8.5)	56.1	70.5	78.0

a Total physical activity level was categorized based on age- (<50, 50–59, and ≥60 years) and sex-specific tertile cutoff points. BMI, body mass index; WC, waist circumference.

### Blood pressure trajectories and change

**FIGURE 1:**
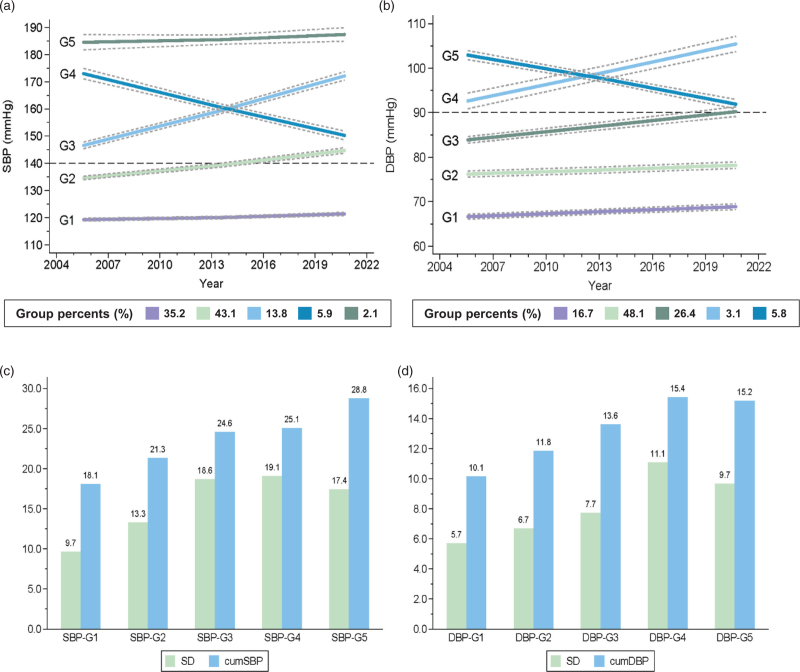
Blood pressure trajectory groups and corresponding blood pressure change indices. (a) SBP trajectory groups; (b) DBP trajectory groups; the solid lines indicate the expected trend; and the gray dashed lines form a 95% confidence interval on the estimated probabilities of group membership. (c) Average SD and cumSBP in SBP trajectory groups; (d) average SD and cumDBP in DBP trajectory groups. CumDBP, cumulative diastolic blood pressure (100 mmHg × year); cumSBP, cumulative systolic blood pressure (100 mmHg × year); SD, standard deviation (mmHg).

The SBP trajectory was grouped into two low-risk groups, SBP-G1 (normal, low-growth) and SBP-G2 (elevated, low-growth), and three high-risk groups, SBP-G3 (high SBP, high-growth), SBP-G4 (high SBP, decrease), and SBP-G5 (extreme high SBP, low-growth) (Fig. 1a). The DBP trajectory was grouped into three low-risk groups, DBP-G1 (optimal, low-growth), DBP-G2 (normal, low-growth), and DBP-G3 (elevated, low-growth), and two high-risk groups, DBP-G4 (high DBP, high-growth) and DBP-G5 (extreme high DBP, decrease) (Fig. 1b). Three BPC measures, SD, CV, and ARV, were highly correlated with each other, while cumBP had high correlations with mean blood pressure (Table S2, Supplemental Digital Content). The correlations among other measures were low. SD and cumBP increased with the risk level of blood pressure trajectory groups (Fig. 1c and d).

### Relationship between baseline characteristics and blood pressure change measures

Table 2 presents the covariate-adjusted differences in SD and cumBP by baseline characteristics of participants. Most baseline characteristics were associated with cumSBP and cumDBP. The cumBP increased with BMI and WC but decreased with total physical activity level. There was a higher cumBP among rural residents, less educated participants, alcohol drinkers (especially heavy drinkers of ≥60 g pure alcohol per day), those who did not eat red meat often, and those with overweight or obesity, while a lower cumBP among smokers. Baseline characteristics related to SD, CV, and ARV were education, alcohol drinking, BMI, WC, and body shape (Table 2 and Table S3, Supplemental Digital Content). Of those, the associations had consistent directions with those of cumBP. The further analyses in hypertensive and normotensive participants showed consistent associations of those baseline characteristics with BPC, but the effect estimates were stronger in hypertensive than in normotensive participants (Tables S4–S5, Supplemental Digital Content). The analyses in participants without antihypertensive treatment also showed consistent associations of those baseline characteristics with BPC (Table S6, Supplemental Digital Content). The baseline characteristics of participants with hypertension, normotension, and without antihypertensive treatment were shown in Table S7, Supplemental Digital Content.

**TABLE 2:** The adjusted differences in SD and cumBP by baseline population characteristics

	SBP		DBP	
	SD	cumSBP	SD	cumDBP
Mean values for all participants	13.2	2099.6	7.1	1233.3
Age, per 10 years	1.6 (1.4, 1.8)	116.9 (111.0, 122.7)	−0.1 (−0.1, 0.0)	−1.1 (−4.4, 2.1)
Sex				
Women	Reference	Reference	Reference	Reference
Men	−1.3 (−1.7, −0.8)	−11.5 (−27.5, 4.5)	0.2 (0.0, 0.5)	24.6 (15.6, 33.6)
Residence				
Urban	Reference	Reference	Reference	Reference
Rural	0.0 (−0.3, 0.3)	63.6 (52.6, 74.7)	0.2 (−0.0, 0.3)	42.1 (35.9, 48.3)
Education				
College or university	Reference	Reference	Reference	Reference
Middle or high school	1.3 (0.6, 2.1)	39.8 (12.6, 67.1)	0.5 (0.2, 0.9)	13.0 (−2.3, 28.3)
Primary school or below	1.6 (0.8, 2.4)	48.1 (19.2, 77.0)	0.7 (0.3, 1.1)	14.1 (−2.1, 30.3)
Smoking				
Never	Reference	Reference	Reference	Reference
Former	−0.2 (−0.8, 0.5)	−21.4 (−46.3, 3.6)	−0.1 (−0.4, 0.3)	−9.0 (−23.0, 5.0)
Current, cigarettes (or equivalent)/day				
< 20	0.3 (−0.2, 0.8)	−38.3 (−57.1, −19.4)	0.0 (−0.3, 0.3)	−24.5 (−35.1, −14.0)
≥ 20	0.5 (0.0, 1.0)	−28.4 (−47.2, −9.6)	0.1 (−0.2, 0.4)	−19.1 (−29.7, −8.6)
Alcohol drinking				
Less than weekly	Reference	Reference	Reference	Reference
Former	0.7 (0.0, 1.4)	48.9 (23.1, 74.8)	0.4 (0.0, 0.8)	30.0 (15.5, 44.5)
Weekly	0.3 (−0.3, 0.9)	54.3 (33.3, 75.4)	0.6 (0.3, 0.9)	33.8 (21.9, 45.6)
Daily, g/day (pure alcohol)				
< 30	0.8 (−0.0, 1.7)	44.0 (11.8, 76.2)	0.3 (−0.2, 0.7)	37.9 (19.8, 55.9)
30–59	1.1 (0.3, 1.9)	54.2 (25.4, 83.0)	0.6 (0.2, 1.0)	39.3 (23.1, 55.4)
≥ 60	1.3 (0.6, 2.1)	86.7 (60.7, 112.6)	0.7 (0.4, 1.1)	51.7 (37.1, 66.2)
Fresh vegetable consumption				
Daily	Reference	Reference	Reference	Reference
Nondaily	−0.2 (−0.8, 0.4)	23.4 (1.0, 45.8)	−0.3 (−0.6, 0.1)	11.2 (−1.4, 23.8)
Fresh fruit consumption				
Daily	Reference	Reference	Reference	Reference
Nondaily	0.3 (−0.1, 0.7)	−2.6 (−17.2, 11.9)	0.1 (−0.1, 0.3)	−4.1 (−12.3, 4.1)
Red meat consumption				
Daily	Reference	Reference	Reference	Reference
Weekly	0.1 (−0.2, 0.4)	2.0 (−10.1, 14.1)	0.1 (−0.1, 0.2)	−0.1 (−6.9, 6.7)
Less than weekly	0.2 (−0.2, 0.7)	29.4 (12.0, 46.8)	0.3 (0.0, 0.5)	11.1 (1.3, 20.8)
Total physical activity, per 4 MET-h/day^a^	−0.03 (−0.07, 0.01)	−2.73 (−4.15, −1.31)	0.00 (−0.02, 0.02)	−1.57 (−2.37, −0.77)
BMI, per 5 kg/m^2^	0.7 (0.5, 0.9)	127.8 (120.7, 134.9)	0.3 (0.2, 0.4)	75.7 (71.7, 79.7)
WC, per 10 cm	0.4 (0.3, 0.6)	70.2 (65.0, 75.3)	0.2 (0.1, 0.3)	42.3 (39.4, 45.1)
Body shape				
BMI <18.5	−0.6 (−1.3, 0.0)	−121.6 (−145.9, −97.3)	−0.3 (−0.7, 0.0)	−62.3 (−76.0, −48.6)
BMI 18.5–23.9, WC <90/85	Reference	Reference	Reference	Reference
BMI 18.5–23.9, WC ≥90/85	−0.4 (−1.7, 0.8)	62.2 (15.9, 108.5)	−0.4 (−1.0, 0.3)	35.0 (9.0, 61.0)
BMI 24.0–27.9, WC <90/85	0.8 (0.5, 1.1)	111.6 (100.3, 122.9)	0.2 (0.0, 0.3)	68.1 (61.8, 74.4)
BMI 24.0–27.9, WC ≥90/85	0.6 (0.2, 1.0)	110.4 (94.6, 126.2)	0.2 (0.0, 0.5)	69.0 (60.2, 77.9)
BMI ≥28.0, WC <90/85	1.4 (0.3, 2.4)	172.3 (133.2, 211.4)	0.3 (−0.3, 0.8)	119.2 (97.2, 141.1)
BMI ≥28.0, WC ≥90/85	0.9 (0.5, 1.4)	198.7 (181.8, 215.6)	0.5 (0.3, 0.8)	116.2 (106.7, 125.7)

The table presents the adjusted difference (95% confidence interval) in SD and cumBP for categorical variables compared with the reference group and adjusted SD and cumBP change (95% CI) per specified unit for continuous variables. Please refer to the method section for detailed covariate adjustment.

a The 4 MET-h/day is equivalent to about 1 h of moderate physical activity per day. BMI, body mass index; cumBP, cumulative blood pressure; cumDBP, cumulative diastolic blood pressure (mmHg × year); cumSBP, cumulative systolic blood pressure (mmHg × year); MET-h/d, metabolic equivalent task-hour/day; SD, standard deviation (mmHg); WC, waist circumference.

### Relationship between baseline characteristics and blood pressure trajectories

Tables 3, S8, Supplemental Digital Content, and Table S9, Supplemental Digital Content present the covariate-adjusted associations of baseline characteristics with SBP, DBP, and combined blood pressure trajectory groups, respectively. The elderly aged 60 and over were more likely to have trajectories of SBP-G2 (elevated, low-growth), SBP-G3 (high SBP, high-growth), or SBP-G4 (high SBP, decrease) but less likely to have trajectories of DBP-G3 (elevated, low-growth) or DBP-G4 (high DBP, high-growth). The corresponding RRRs (95%CIs) were 1.41 (1.25, 1.59), 1.24 (1.04, 1.47), 2.48 (1.97, 3.11), 0.70 (0.59, 0.83) and 0.58 (0.41, 0.81) compared with participants aged <50 (Tables 3 and S8, Supplemental Digital Content). For the combined blood pressure trajectories, the elderly aged 60 and over were more likely to have the isolated SBP high-risk trajectory or the isolated DBP high-risk trajectory but less likely to have both SBP and DBP high-risk trajectories (Table S9, Supplemental Digital Content). Male participants were more likely to have various DBP high-risk trajectories.

**TABLE 3:** Association between baseline characteristics and SBP trajectory groups

	G2	G3	G4	G5
Age, years				
<50	Reference	Reference	Reference	Reference
50–59	1.16 (1.06, 1.27)	1.12 (0.99, 1.27)	1.90 (1.59, 2.27)	1.21 (0.92, 1.59)
≥60	1.41 (1.25, 1.59)	1.24 (1.04, 1.47)	2.48 (1.97, 3.11)	1.20 (0.83, 1.75)
Sex				
Women	Reference	Reference	Reference	Reference
Men	1.13 (1.00, 1.29)	0.92 (0.76, 1.10)	0.95 (0.73, 1.24)	0.44 (0.27, 0.73)
Residence				
Urban	Reference	Reference	Reference	Reference
Rural	1.05 (0.96, 1.14)	1.14 (1.01, 1.29)	0.99 (0.83, 1.17)	1.62 (1.20, 2.17)
Education				
College or university	Reference	Reference	Reference	Reference
Middle or high school	1.20 (0.97, 1.49)	1.41 (1.03, 1.94)	1.52 (0.90, 2.58)	2.37 (0.83, 6.81)
Primary school or below	1.17 (0.93, 1.47)	1.39 (0.99, 1.94)	1.47 (0.85, 2.53)	3.13 (1.06, 9.22)
Smoking				
Never	Reference	Reference	Reference	Reference
Former	1.01 (0.82, 1.24)	0.90 (0.67, 1.20)	0.68 (0.45, 1.03)	1.70 (0.90, 3.22)
Current, cigarettes (or equivalent)/day				
< 20	0.83 (0.71, 0.97)	0.72 (0.57, 0.90)	0.71 (0.52, 0.98)	0.72 (0.39, 1.35)
≥ 20	0.86 (0.74, 1.00)	0.84 (0.67, 1.04)	0.78 (0.57, 1.06)	0.78 (0.42, 1.45)
Alcohol drinking				
Less than weekly	Reference	Reference	Reference	Reference
Former	0.99 (0.80, 1.23)	1.13 (0.83, 1.53)	1.75 (1.21, 2.54)	2.51 (1.40, 4.50)
Weekly	1.16 (0.97, 1.38)	1.46 (1.15, 1.85)	1.42 (1.00, 2.03)	2.17 (1.24, 3.79)
Daily, g/day (pure alcohol)				
< 30	0.94 (0.72, 1.21)	1.06 (0.73, 1.54)	0.72 (0.39, 1.34)	1.87 (0.83, 4.19)
30–59	1.23 (0.97, 1.55)	1.30 (0.92, 1.84)	1.78 (1.15, 2.77)	0.60 (0.14, 2.52)
≥ 60	1.56 (1.26, 1.94)	2.05 (1.52, 2.76)	1.98 (1.29, 3.05)	1.23 (0.43, 3.53)
Fresh vegetable consumption				
Daily	Reference	Reference	Reference	Reference
Nondaily	1.05 (0.88, 1.27)	1.17 (0.90, 1.52)	1.41 (1.02, 1.96)	0.90 (0.49, 1.64)
Fresh fruit consumption				
Daily	Reference	Reference	Reference	Reference
Nondaily	1.09 (0.97, 1.23)	1.25 (1.05, 1.48)	1.37 (1.07, 1.74)	1.18 (0.80, 1.75)
Red meat consumption				
Daily	Reference	Reference	Reference	Reference
Weekly	0.98 (0.89, 1.08)	0.98 (0.85, 1.13)	1.04 (0.85, 1.27)	0.97 (0.70, 1.35)
Less than weekly	0.98 (0.85, 1.13)	1.07 (0.88, 1.30)	1.16 (0.88, 1.53)	1.24 (0.81, 1.91)
Total physical activity^a^				
Low	Reference	Reference	Reference	Reference
Medium	0.98 (0.90, 1.08)	1.02 (0.90, 1.16)	0.90 (0.75, 1.08)	0.92 (0.69, 1.21)
High	1.03 (0.93, 1.14)	1.02 (0.89, 1.17)	0.99 (0.82, 1.21)	0.73 (0.53, 1.00)
BMI				
<18.5 kg/m^2^	Reference	Reference	Reference	Reference
18.5–23.9 kg/m^2^	1.81 (1.50, 2.18)	2.78 (1.93, 4.00)	3.55 (1.92, 6.57)	12.58 (1.75, 90.57)
24.0–27.9 kg/m^2^	3.35 (2.77, 4.07)	7.26 (5.03, 10.47)	11.51 (6.20, 21.37)	48.60 (6.76, 349.57)
≥28.0 kg/m^2^	5.04 (4.00, 6.35)	13.80 (9.31, 20.46)	29.59 (15.61, 56.09)	103.42 (14.18, 754.12)
WC (men/women)				
<85/80 cm	Reference	Reference	Reference	Reference
85–90/80–85 cm	1.69 (1.53, 1.88)	2.27 (1.98, 2.60)	2.80 (2.32, 3.38)	3.55 (2.66, 4.74)
≥90/85 cm	2.26 (2.03, 2.52)	3.31 (2.89, 3.79)	4.64 (3.88, 5.55)	5.19 (3.93, 6.84)
Body shape				
BMI <18.5	0.56 (0.46, 0.67)	0.36 (0.25, 0.52)	0.29 (0.16, 0.53)	0.08 (0.01, 0.59)
BMI 18.5–23.9, WC <90/85	Reference	Reference	Reference	Reference
BMI 18.5–23.9, WC ≥90/85	1.27 (0.87, 1.84)	1.50 (0.89, 2.52)	2.03 (1.06, 3.89)	1.75 (0.62, 5.00)
BMI 24.0–27.9, WC <90/85	1.77 (1.61, 1.94)	2.50 (2.21, 2.84)	3.14 (2.62, 3.76)	3.70 (2.78, 4.92)
BMI 24.0–27.9, WC ≥90/85	2.19 (1.91, 2.51)	3.08 (2.58, 3.67)	3.93 (3.10, 4.98)	4.57 (3.19, 6.54)
BMI ≥28.0, WC <90/85	2.30 (1.60, 3.30)	3.97 (2.59, 6.08)	7.00 (4.15, 11.83)	3.92 (1.50, 10.23)
BMI ≥28.0, WC ≥90/85	2.89 (2.46, 3.40)	5.19 (4.28, 6.31)	8.77 (6.89, 11.16)	9.18 (6.43, 13.10)

The table presents the adjusted RRR (95% CI), with G1 being the reference group. Please refer to the method section for detailed covariate adjustment.

a Total physical activity level was categorized based on age- (<50, 50–59, and ≥60 years) and sex-specific tertile cutoff points. BMI, body mass index; G1, normal, low-growth (reference); G2, elevated, low-growth; G3, high SBP, high-growth; G4, high SBP, decrease; G5, extreme high SBP, low-growth; RRR, relative risk ratio; SBP, systolic blood pressure; WC, waist circumference.

Compared with nonsmokers, participants who smoked <20 cigarettes (or equivalent) per day were less likely to have the both SBP and DBP high-risk trajectories, of which the RRRs (95% CIs) was 0.66 (0.50, 0.86). Alcohol drinking, especially heavy drinking of ≥60 g pure alcohol per day and drinking cessation, was associated with several high-risk trajectories of SBP and DBP. Compared with less than weekly drinking, the RRRs (95% CIs) of ≥60 g pure alcohol per day drinking were 1.57 (1.11, 2.23) for the both SBP and DBP high-risk trajectories.

Both BMI and WC were independently associated with all high-risk blood pressure trajectories. Compared with participants who had BMI of 18.5–23.9 kg/m^2^ and WC<90/85 cm, those with BMI ≥28.0 kg/m^2^ and WC≥90/85 cm had higher risk of SBP-G3, SBP-G4, SBP-G5, DBP-G4, and DBP-G5, with the RRRs (95% CIs) of 5.19 (4.28, 6.31), 8.77 (6.89, 11.16), 9.18 (6.43, 13.10), 10.63 (7.35, 15.37), and 13.76 (10.10, 18.75), respectively. Larger association estimates were observed for the relation of overweight, general, and central obesity with the both SBP and DBP high-risk trajectories than the isolated SBP high-risk trajectory or the isolated DBP high-risk trajectory.

## DISCUSSION

In this cohort study of more than 15 000 Chinese adults followed up for 15 years, we found that most traditional CVD risk factors had important influences on cumBP and blood pressure trajectories. By contrast, the differences in SD, CV, and ARV by factors of interest were statistically significant but weak. Lifestyle factors, such as alcohol drinking, not eating red meat often (especially hardly eating red meat), and general and central obesity, were associated with increased long-term cumBP and adverse blood pressure trajectories. The urban/rural residence and education also had relations with long-term cumBP and adverse blood pressure trajectories, which could not be explained fully by the differences in lifestyle.

A study based on the third National Health and Nutrition Examination Survey (NHANES III) showed that BMI and WC were associated with higher short-term variability of SBP within one month but not with DBP [21]. After adjustment for age, race, and smoking, the SBP-SD in overweight (BMI of 25–29.9 kg/m^2^) and obese (≥30 kg/m^2^) participants rose by 0.06 mmHg and 0.25 mmHg compared with participants of normal weight (18.5–24.9 kg/m^2^). Central obesity was associated with increased SBP-SD by 0.31 mmHg. Another study used data from the China Health and Nutrition Survey (CHNS) between 1989 and 2009 to identify 20-year blood pressure trajectories in normotensive adults. Compared with stable low blood pressure levels, the risk of moderate to high increasing trajectories increased by 8–11% for SBP and 5–8% for DBP per 1 cm larger WC, respectively [22]. The present study included both normotensive and hypertensive participants. We found that the SD, ARV, cumBP, and occurrence of high-risk trajectories in both SBP and DBP increased with higher BMI or WC. Participants with overweight, general, and central obesity were more likely to be with both SBP and DBP high-risk trajectories.

Alcohol drinking has been associated with elevated blood pressure, and the effect increases with higher alcohol consumption [23,24]. However, there is no research assessing the relationship between alcohol drinking and long-term BPC or blood pressure trajectories yet. In our study, compared with less than weekly drinkers, both cumSBP and cumDBP increased with higher daily alcohol intake. Alcohol drinking, especially moderate or above levels of intake, was strongly associated with several high-risk trajectories of SBP and DBP. The heavy drinkers were more likely to have the both SBP and DBP high-risk trajectories. Additionally, we found that the following 15-year cumBP and blood pressure trajectories in baseline former drinkers resembled those in moderate to heavy drinkers after adjusting for potential confounders. One possible explanation is that a certain number of former drinkers had been heavy drinkers but stopped due to illness, which was similar to the fact that about half of the former smokers among the CKB population had quitted because they were ill [25]. The persistent excessive alcohol use possibly had irreversible effects on the adverse development of blood pressure, which warrants more studies. Also, some baseline abstainers may relapse during the follow-up period, which we could not assess because we only collected drinking status once at baseline.

The relationship between dietary red meat and blood pressure remains inconsistent. A study found that SBP/DBP increased with higher unprocessed red meat consumption (0.70/0.55 mmHg per 25 g/1000 kcal) in Western participants, while consumption of 25 g/1000 kcal unprocessed red meat was not related to blood pressure in East Asian participants [26]. The CKB baseline survey collected the intake frequency of red meat and its products, but processed red meat constituted only 3.1% of the total red meat intake in China during that period [27]. As a limitation, we did not collect the daily amount of red meat. However, in our population, a certain proportion of participants rarely ate red meat, which differed from those who ate red meat daily but at a low level. After controlling for socioeconomic status (SES) and other lifestyle factors, we observed that participants who rarely ate red meat had a higher cumSBP and cumDBP compared with those who ate daily. Previous studies have investigated the association between vegetarian diets and blood pressure. A meta-analysis indicated that vegetarian diets were associated with significantly lower SBP/DBP compared with omnivorous diets in seven clinical trials (−4.8/−2.2 mmHg) and 32 observational studies (−6.9/−4.7 mmHg) [28]. The vegetarian and vegan diets prevalent in Western populations are more likely to be a choice of dietary pattern, with essential nutrients provided by alternative foods or dietary supplements. No matter whether for economic reasons or voluntary choices, the overall diet quality for those who rarely ate red meat in the CKB should be poor at baseline, which may partly explain the unfavorable blood pressure change.

The chronic effects of cigarette smoking on blood pressure remain mixed. Two studies from Japan found that smoking was associated with reduced 5-year changes in blood pressure and incidence of hypertension [29] or temporal decrease in blood pressure [30]. The Coronary Artery Risk Development in Young Adults (CARDIA) study showed that consistent smokers had similar SBP but lower DBP (β = −2.27 mmHg) compared with never smokers in Whites [31]. Neither SBP nor DBP differed between consistent smokers and never smokers in Blacks. In the present study, smokers, particularly those who smoked <20 cigarettes (or equivalent) per day, had a lower cumBP and were less likely to have high-risk blood pressure trajectories than nonsmokers. Although it has been suggested that a lower BMI among current smokers may contribute to the negative association between chronic smoking and blood pressure, we demonstrated the aforementioned negative association even after adjusting for BMI and other lifestyle factors. The mechanisms linking smoking to long-term BPC are poorly understood. On the one hand, it is likely that the vasodilatory effects of cotinine, a metabolite of nicotine [32], and carbon monoxide [33] contribute to a lower total vascular resistance among smokers so that the blood pressure is unable to rise easily. On the other hand, psychophysiological stress responsiveness may be inhibited in chronic smokers, leading to relatively stable blood pressure [34].

Previous studies from the United States [35,36] and Korea [37] demonstrated that lower SES, especially poor educational status, was associated with an increased risk of incident hypertension among middle-aged and older adults. We observed a higher cumBP and occurrence of several high-risk SBP trajectories in rural residents and less educated participants after adjusting for lifestyle factors, suggesting that the observed differences in participants with different SES cannot be fully explained by those measured lifestyle factors.

To the best of our knowledge, this is the first study to comprehensively explore the associations of sociodemographic characteristics and lifestyle factors with long-term BPC and trajectories. Strengths of our study include its large sample size, long-term follow-up, and broad geographic distribution and diversity in the SES characteristics of our population. Limitations of the present study also warrant consideration. First, blood pressure measurements were available at only three time points within fifteen years. More frequent fluctuations in blood pressure during visits cannot be captured. Second, due to the limited information on antihypertensive drugs, we cannot perform a more in-depth analysis on drug types and numbers. Third, we investigated dietary habits using a short qualitative food frequency questionnaire and asked about the weekly intake frequency of some conventional food groups instead of the daily amount of consumption.

In this cohort study of Chinese adults with three visits in 15 years, we identified the associations of lifestyle factors, including adiposity and alcohol drinking, with unfavorable long-term BPC and blood pressure trajectories. In previous studies of the CKB population, adiposity and alcohol drinking have been associated with CVD [38–40]. When putting these results together, these factors may increase CVD risk (especially ischemic and hemorrhagic stroke risk), at least in part, by unfavorable long-term BPC. Additionally, findings about unfavorable long-term cumBP in participants who rarely ate red meat may explain the inverse associations between intakes of red meat and the risk of intracerebral hemorrhage observed in the CKB population [41]. Further studies are warranted to confirm our findings and investigate the potential mechanisms that underlie the observed associations.

## ACKNOWLEDGEMENTS

The most important acknowledgment is to the participants in the study and the members of the survey teams in each of the 10 regional centers, as well as to the project development and management teams based at Beijing, Oxford and the 10 regional centers.

Sources of funding: This work was supported by National Natural Science Foundation of China (82192904, 82388102, 82192900). The CKB baseline survey and the first re-survey were supported by a grant from the Kadoorie Charitable Foundation in Hong Kong. The long-term follow-up is supported by grants from the UK Wellcome Trust (212946/Z/18/Z, 202922/Z/16/Z, 104085/Z/14/Z, 088158/Z/09/Z), grants from the National Key R&D Program of China (2016YFC0900500), National Natural Science Foundation of China (81390540, 91846303, 81941018), and Chinese Ministry of Science and Technology (2011BAI09B01). The funders had no role in the study design, data collection, data analysis and interpretation, writing of the report, or the decision to submit the article for publication.

Authors’ contributions: J.L. conceived and designed the study. L.L., Z.C., and J.C., members of the China Kadoorie Biobank Steering Committee, designed and supervised the whole study, obtained funding, and, together with C.Y., D.S., Y.P., P.P., H.D., L.Y., Y.C., X.Y. and X.C. acquired the data. Y.Z. and Q.S. analyzed the data. Y.Z. drafted the manuscript. J.L. contributed to the interpretation of the results and critical revision of the manuscript for important intellectual content. All authors reviewed and approved the final manuscript. J.L. is the study guarantor.

Data sharing: The access policy and procedures are available at www.ckbiobank.org.

### Conflicts of interest

There are no conflicts of interest.

## Supplementary Material

Supplemental Digital Content
