# Supplementary material for: Associations of traditional cardiovascular risk factors with 15-year blood pressure change and trajectories in Chinese adults: a prospective cohort study
Source: J Hypertens. 2024 Mar 22;42(8):1340–9. doi: 10.1097/HJH.0000000000003717 (PMC7616121; doi:10.1097/HJH.0000000000003717)
Supplement: Supplemental Digital Content [file jhype-42-1340-s001.docx]

**Supplemental Material**

Supplemental material for the manuscript entitled “Associations of traditional cardiovascular risk factors with 15-year blood pressure change and trajectories in Chinese adults: a prospective cohort study”

**Contents**

[**Members of the China Kadoorie Biobank collaborative group:** 1](#_Toc155867864)

[**Supplementary methods** 2](#_Toc155867865)

[**Assessment of lifestyle factors** 2](#_Toc155867866)

[**Coefficient of variation (CV) and average real variability (ARV) calculations** 2](#_Toc155867867)

[**Trajectory modeling approach** 2](#_Toc155867868)

[**References** 3](#_Toc155867869)

[**Table S1.** The distribution of anti-hypertensive drug types and numbers in participants with hypertension 4](#_Toc155867870)

[**Table S2.** Spearman's rank correlation coefficients between metrics of BPC and mean BP 5](#_Toc155867871)

[**Table S3.** The adjusted differences in CV and ARV by baseline population characteristics 6](#_Toc155867872)

[**Table S4.** The adjusted differences in SBP change indices by baseline characteristics in hypertensive and normotensive participants 8](#_Toc155867873)

[**Table S5.** The adjusted differences in DBP change indices by baseline characteristics in hypertensive and normotensive participants 10](#_Toc155867874)

[**Table S6.** The adjusted differences in SBP and DBP change indices by baseline characteristics in participants without antihypertensive treatment 12](#_Toc155867875)

[**Table S7.** The baseline characteristics of participants with hypertension, normotension, and without anti-hypertensive treatment 14](#_Toc155867876)

[**Table S8.** Association between baseline characteristics and DBP trajectory groups 15](#_Toc155867877)

[**Table S9.** Association between baseline characteristics and combined blood pressure trajectories 17](#_Toc155867878)

**Members of the China Kadoorie Biobank collaborative group:**

**International Steering Committee:** Junshi Chen, Zhengming Chen (PI), Robert Clarke, Rory Collins, Liming Li (PI), Jun Lv, Richard Peto, Robin Walters.

**International Co-ordinating Centre, Oxford:** Daniel Avery, Maxim Barnard, Derrick Bennett, Lazaros Belbasis, Ruth Boxall, Ka Hung Chan, Yiping Chen, Zhengming Chen, Charlotte Clarke, Johnathan Clarke; Robert Clarke, Huaidong Du, Ahmed Edris Mohamed, Hannah Fry, Simon Gilbert, Pek Kei Im, Andri Iona, Maria Kakkoura, Christiana Kartsonaki, Hubert Lam, Kuang Lin, James Liu, Mohsen Mazidi, Iona Millwood, Sam Morris, Qunhua Nie, Alfred Pozarickij, Maryanm Rahmati, Paul Ryder, Saredo Said, Dan Schmidt, Becky Stevens, Iain Turnbull, Robin Walters, Baihan Wang, Lin Wang, Neil Wright, Ling Yang, Xiaoming Yang, Pang Yao.

**National Co-ordinating Centre, Beijing:** Xiao Han, Can Hou, Qingmei Xia, Chao Liu, Jun Lv, Pei Pei, Dianjianyi Sun, Canqing Yu, Lang Pan.

**10 Regional Co-ordinating Centres:**

**Qingdao CDC:** Zengchang Pang, Ruqin Gao, Shanpeng Li, Haiping Duan, Shaojie Wang, Yongmei Liu, Ranran Du, Yajing Zang, Liang Cheng, Xiaocao Tian, Hua Zhang, Yaoming Zhai, Feng Ning, Xiaohui Sun, Feifei Li. **Licang CDC:** Silu Lv, Junzheng Wang, Wei Hou. **Heilongjiang Provincial CDC:** Wei Sun, Shichun Yan, Xiaoming Cui. **Nangang CDC:** Chi Wang, Zhenyuan Wu,Yanjie Li, Quan Kang. **Hainan Provincial CDC:** Huiming Luo, Tingting Ou. **Meilan CDC:** Xiangyang Zheng, Zhendong Guo, Shukuan Wu, Yilei Li, Huimei Li. **Jiangsu Provincial CDC:** Ming Wu, Yonglin Zhou, Jinyi Zhou, Ran Tao, Jie Yang, Jian Su. **Suzhou CDC:** Fang Liu, Jun Zhang, Yihe Hu, Yan Lu, Liangcai Ma, Aiyu Tang, Shuo Zhang, Jianrong Jin, Jingchao Liu. **Guangxi Provincial CDC:** Mei Lin, Zhenzhen Lu. **Liuzhou CDC:** Lifang Zhou, Changping Xie, Jian Lan,Tingping Zhu,Yun Liu, Liuping Wei, Liyuan Zhou, Ningyu Chen, Yulu Qin, Sisi Wang. **Sichuan Provincial CDC:** Xianping Wu, Ningmei Zhang, Xiaofang Chen, Xiaoyu Chang. **Pengzhou CDC:** Mingqiang Yuan, Xia Wu, Xiaofang Chen, Wei Jiang, Jiaqiu Liu, Qiang Sun. **Gansu Provincial CDC:** Faqing Chen, Xiaolan Ren, Caixia Dong. **Maiji CDC:** Hui Zhang, Enke Mao, Xiaoping Wang, Tao Wang, Xi zhang. **Henan Provincial CDC:** Kai Kang, Shixian Feng, Huizi Tian, Lei Fan. **Huixian CDC:** XiaoLin Li, Huarong Sun, Pan He, Xukui Zhang. **Zhejiang Provincial CDC:** Min Yu, Ruying Hu, Hao Wang. **Tongxiang CDC:** Xiaoyi Zhang, Yuan Cao, Kaixu Xie, Lingli Chen, Dun Shen. **Hunan Provincial CDC:** Xiaojun Li, Donghui Jin, Li Yin, Huilin Liu, Zhongxi Fu. **Liuyang CDC:** Xin Xu, Hao Zhang, Jianwei Chen,Yuan Peng, Libo Zhang, Chan Qu.

**Supplementary methods**

**Assessment** **of lifestyle factors**

In the interviewer-administered questionnaire of the CKB study, ever smokers were asked for frequency, type, and the amount of tobacco smoked per day, and former smokers were also asked for years since stopping and the reason for quitting smoking^[1]^. Participants who drank at least once a week were asked for the types of alcoholic beverages consumed and the amount drunk on a typical drinking day in the prior 12 months^[2]^. For physical activity, participants were asked about the usual type and duration of activities in occupational, commuting, domestic, and leisure-time related domains in the past 12 months. Total physical activity was calculated by multiplying the metabolic equivalent of task (MET) value of each type of activity and the hours spent on that activity per day and then summarizing the MET-hours for all activities^[3]^. Qualitative habitual intakes of 12 conventional food groups in the past 12 months were assessed using a validated food frequency questionnaire^[4]^.

**Coefficient of variation (CV) and average real variability (ARV) calculations**

The CV of blood pressure was calculated as SD divided by mean SBP or mean DBP. ARV was the average absolute difference between consecutive blood pressure measurements and was calculated using the following formula:

$$ARV=\frac{\left| {BP}_{2}-{BP}_{1} \right|+\left| {BP}_{3}-{BP}_{2} \right|}{2}$$

**Trajectory modeling approach**

Given that the blood pressure of our participants was approximately normally distributed, we estimated parameters for continuous and normal distribution data using the censored normal model, with follow-up year as the time scale and age as a time-varying covariate. The evaluation of linear or quadratic terms was based on the 0.05 significant level, starting with the highest polynomial. We initiated models with 3, 4 and 5 trajectories, respectively. Then chose the optimal number by comparing the BIC for each number of trajectories. Finally, the model with 5 trajectories identified fit best.

**References**

1. Liu X, Bragg F, Yang L, Kartsonaki C, Guo Y, Du H, et al. Smoking and smoking cessation in relation to risk of diabetes in Chinese men and women: a 9-year prospective study of 0·5 million people. Lancet Public Health 2018; 3:e167-e76.

2. Millwood IY, Li L, Smith M, Guo Y, Yang L, Bian Z, et al. Alcohol consumption in 0.5 million people from 10 diverse regions of China: prevalence, patterns and socio-demographic and health-related correlates. Int J Epidemiol 2013; 42:816-27.

3. Du H, Bennett D, Li L, Whitlock G, Guo Y, Collins R, et al. Physical activity and sedentary leisure time and their associations with BMI, waist circumference, and percentage body fat in 0.5 million adults: the China Kadoorie Biobank study. Am J Clin Nutr 2013; 97:487-96.

4. Qin C, Guo Y, Pei P, Du H, Yang L, Chen Y, et al. The Relative Validity and Reproducibility of Food Frequency Questionnaires in the China Kadoorie Biobank Study. Nutrients 2022; 14:794.

**Table S1.** The distribution of anti-hypertensive drug types and numbers in participants with hypertension

|  | 2004–08 baseline  (n= 5,324) | 2013–14 resurvey  (n= 7,762) | 2020–21 resurvey  (n= 9,478) |
| --- | --- | --- | --- |
| Participants with anti-hypertensive treatment^*^ | 1,512 (28.4) | 4,074 (52.5) | 6,577 (69.4) |
| **Drug type^**^** |  |  |  |
| Angiotensin-converting enzyme inhibitor | 160 (10.6) | 302 (7.4) | 283 (4.3) |
| Beta blocker | 100 (6.6) | 388 (9.5) | 370 (5.6) |
| Diuretic | 20 (1.3) | 114 (2.8) | 348 (5.3) |
| Calcium channel blocker | 341 (22.6) | 787 (19.3) | 2,248 (34.2) |
| Angiotensin receptor blocker | N/A | 362 (8.9) | 1,205 (18.3) |
| Other anti-hypertensive drugs | N/A | 2,356 (57.8) | 2,959 (45.0) |
| Unknown | 985 (65.1) | 668 (16.4) | 567 (8.6) |
| **Drug number^**^** |  |  |  |
| 1 | 438 (29.0) | 2,693 (66.1) | 4,812 (73.2) |
| 2 | 84 (5.6) | 563 (13.8) | 1,014 (15.4) |
| 3 | 5 (0.3) | 116 (2.9) | 166 (2.5) |
| 4 | N/A | 29 (0.7) | 16 (0.2) |
| 5 | N/A | 4 (0.1) | 1 (0.02) |
| 6 | N/A | 1 (0.02) | 1 (0.02) |

Data was presented as number (%).

^*^Using participants with hypertension as denominator.

^**^Using participants with anti-hypertensive treatment as denominator.

**Table S2.** Spearman's rank correlation coefficients between metrics of BPC and mean BP

|  | Mean | SD | CV | ARV | cumBP |
| --- | --- | --- | --- | --- | --- |
| **SBP** |  |  |  |  |  |
| Mean | 1.000 |  |  |  |  |
| SD | 0.417 | 1.000 |  |  |  |
| CV | 0.221 | 0.974 | 1.000 |  |  |
| ARV | 0.382 | 0.925 | 0.902 | 1.000 |  |
| cumSBP | 0.916 | 0.412 | 0.234 | 0.387 | 1.000 |
| **DBP** |  |  |  |  |  |
| Mean | 1.000 |  |  |  |  |
| SD | 0.225 | 1.000 |  |  |  |
| CV | 0.030 | 0.976 | 1.000 |  |  |
| ARV | 0.212 | 0.917 | 0.894 | 1.000 |  |
| cumDBP | 0.889 | 0.219 | 0.045 | 0.210 | 1.000 |

All P values for Spearman's rank correlation coefficients <0.001.

ARV, average real variability; BP, blood pressure; BPC, blood pressure change; cumDBP, cumulative diastolic blood pressure; cumSBP, cumulative systolic blood pressure; CV, coefficient of variation; SD, standard deviation.

**Table S3.** The adjusted differences in CV and ARV by baseline population characteristics

|  | SBP | |  | DBP | |
| --- | --- | --- | --- | --- | --- |
|  | CV | ARV |  | CV | ARV |
| Mean values for all participants | 9.5 | 16.0 |  | 8.8 | 8.6 |
| Age, per 10 years | 0.7 (0.6, 0.8) | 2.1 (1.9, 2.3) |  | 0.0 (-0.1, 0.1) | 0.1 (0.0, 0.2) |
| Sex |  |  |  |  |  |
| Women | Reference | Reference |  | Reference | Reference |
| Men | -0.9 (-1.2, -0.6) | -1.4 (-1.9, -0.8) |  | 0.1 (-0.1, 0.4) | 0.3 (0.0, 0.6) |
| Residence |  |  |  |  |  |
| Urban | Reference | Reference |  | Reference | Reference |
| Rural | 0.0 (-0.2, 0.2) | -0.6 (-1.0, -0.2) |  | 0.2 (-0.0, 0.4) | 0.2 (-0.0, 0.4) |
| Education |  |  |  |  |  |
| College or university | Reference | Reference |  | Reference | Reference |
| Middle or high school | 0.8 (0.4, 1.3) | 1.8 (0.8, 2.7) |  | 0.6 (0.1, 1.1) | 0.6 (0.1, 1.1) |
| Primary school or below | 1.1 (0.5, 1.6) | 2.2 (1.1, 3.2) |  | 0.8 (0.3, 1.3) | 0.8 (0.3, 1.3) |
| Smoking |  |  |  |  |  |
| Never | Reference | Reference |  | Reference | Reference |
| Former | -0.1 (-0.5, 0.4) | -0.2 (-1.0, 0.7) |  | -0.1 (-0.5, 0.3) | -0.1 (-0.5, 0.4) |
| Current, cigarettes (or equivalent)/d | |  |  |  |  |
| <20 | 0.4 (0.0, 0.7) | 0.2 (-0.4, 0.9) |  | 0.2 (-0.1, 0.5) | 0.0 (-0.4, 0.3) |
| ≥20 | 0.5 (0.1, 0.8) | 0.4 (-0.3, 1.1) |  | 0.3 (-0.0, 0.6) | 0.0 (-0.3, 0.4) |
| Alcohol drinking |  |  |  |  |  |
| Less than weekly | Reference | Reference |  | Reference | Reference |
| Former | 0.4 (-0.1, 0.8) | 0.5 (-0.4, 1.4) |  | 0.3 (-0.1, 0.8) | 0.4 (-0.1, 0.8) |
| Weekly | 0.0 (-0.4, 0.4) | 0.2 (-0.5, 1.0) |  | 0.5 (0.1, 0.9) | 0.7 (0.3, 1.1) |
| Daily, g/d (pure alcohol) |  |  |  |  |  |
| <30 | 0.5 (-0.1, 1.1) | 0.3 (-0.8, 1.5) |  | 0.2 (-0.3, 0.8) | 0.1 (-0.5, 0.7) |
| 30–59 | 0.7 (0.2, 1.2) | 1.1 (0.1, 2.1) |  | 0.6 (0.1, 1.1) | 0.8 (0.2, 1.3) |
| ≥60 | 0.7 (0.2, 1.2) | 1.7 (0.7, 2.6) |  | 0.7 (0.2, 1.1) | 1.0 (0.5, 1.5) |
| Fresh vegetable consumption |  |  |  |  |  |
| Daily | Reference | Reference |  | Reference | Reference |
| Nondaily | -0.2 (-0.6, 0.2) | -0.6 (-1.4, 0.2) |  | -0.4 (-0.8, -0.0) | -0.3 (-0.7, 0.1) |
| Fresh fruit consumption |  |  |  |  |  |
| Daily | Reference | Reference |  | Reference | Reference |
| Nondaily | 0.1 (-0.2, 0.4) | 0.3 (-0.2, 0.8) |  | 0.0 (-0.2, 0.3) | 0.1 (-0.1, 0.4) |
| Red meat consumption |  |  |  |  |  |
| Daily | Reference | Reference |  | Reference | Reference |
| Weekly | 0.0 (-0.2, 0.2) | 0.2 (-0.2, 0.7) |  | 0.1 (-0.1, 0.3) | 0.2 (-0.1, 0.4) |
| Less than weekly | 0.1 (-0.2, 0.4) | 0.3 (-0.3, 0.9) |  | 0.3 (-0.0, 0.6) | 0.2 (-0.1, 0.5) |
| Total physical activity,  per 4 MET-h/d^*^ | -0.02 (-0.04, 0.01) | 0.00 (-0.05, 0.05) |  | 0.00 (-0.02, 0.03) | 0.00 (-0.02, 0.03) |
| BMI, per 5 kg/m^2^ | 0.0 (-0.1, 0.1) | 1.1 (0.9, 1.3) |  | -0.1 (-0.3, -0.0) | 0.5 (0.4, 0.6) |
| WC, per 10 cm | 0.0 (-0.1, 0.1) | 0.7 (0.5, 0.9) |  | -0.1 (-0.2, -0.0) | 0.3 (0.2, 0.4) |
| Body shape |  |  |  |  |  |
| BMI<18.5 | 0.0 (-0.4, 0.5) | -0.8 (-1.7, 0.0) |  | 0.0 (-0.4, 0.4) | -0.4 (-0.9, 0.0) |
| BMI 18.5–23.9, WC<90/85 | Reference | Reference |  | Reference | Reference |
| BMI 18.5–23.9, WC≥90/85 | -0.6 (-1.4, 0.3) | 0.1 (-1.5, 1.7) |  | -0.8 (-1.6, 0.0) | -0.4 (-1.3, 0.4) |
| BMI 24.0–27.9, WC<90/85 | 0.1 (-0.1, 0.3) | 1.3 (0.9, 1.7) |  | -0.2 (-0.4, 0.0) | 0.4 (0.2, 0.6) |
| BMI 24.0–27.9, WC≥90/85 | -0.1 (-0.4, 0.2) | 0.8 (0.2, 1.3) |  | -0.3 (-0.5, 0.0) | 0.3 (0.0, 0.6) |
| BMI≥28.0, WC<90/85 | 0.4 (-0.3, 1.1) | 1.5 (0.2, 2.9) |  | -0.4 (-1.0, 0.3) | 0.3 (-0.4, 1.0) |
| BMI≥28.0, WC≥90/85 | -0.1 (-0.4, 0.2) | 1.6 (1.0, 2.2) |  | -0.1 (-0.4, 0.2) | 1.0 (0.7, 1.3) |

The table presents the adjusted difference (95% confidence interval) of CV and ARV for categorical variables compared with the reference group and adjusted CV and ARV change (95% CI) per specified unit for continuous variables. Please refer to the method section for detailed covariate adjustment.

^*^The 4 MET-h/d is equivalent to about 1 hour of moderate physical activity per day.

ARV, average real variability (mmHg); BMI, body mass index; CV, coefficient of variation (%); DBP, diastolic blood pressure; MET-h/d, metabolic equivalent task-hour/day; SBP, systolic blood pressure; WC, waist circumference.

**Table S4.** The adjusted differences in SBP change indices by baseline characteristics in hypertensive and normotensive participants

|  | Hypertension (n = 10,751) | | | |  | Normotension (n = 5234) | | | |
| --- | --- | --- | --- | --- | --- | --- | --- | --- | --- |
|  | SD | CV | ARV | cumSBP |  | SD | CV | ARV | cumSBP |
| Mean values | 15.6 | 10.7 | 18.8 | 2246.5 |  | 8.1 | 7.0 | 10.2 | 1798.0 |
| Age, per 10 years | 0.6 (0.4, 0.8) | 0.1 (-0.1, 0.2) | 1.1 (0.8, 1.4) | 80.2 (73.6, 86.7) |  | 0.5 (0.3, 0.7) | 0.4 (0.2, 0.5) | 0.6 (0.4, 0.9) | 20.0 (14.4, 25.7) |
| Sex |  |  |  |  |  |  |  |  |  |
| Women | Reference | Reference | Reference | Reference |  | Reference | Reference | Reference | Reference |
| Men | -1.5 (-2.0, -0.9) | -0.9 (-1.3, -0.6) | -1.6 (-2.3, -0.9) | -26.2 (-43.7, -8.6) |  | -0.5 (-0.9, -0.1) | -0.6 (-0.9, -0.2) | -0.5 (-1.1, 0.1) | 37.9 (23.2, 52.6) |
| Residence |  |  |  |  |  |  |  |  |  |
| Urban | Reference | Reference | Reference | Reference |  | Reference | Reference | Reference | Reference |
| Rural | 0.0 (-0.4, 0.4) | 0.0 (-0.3, 0.2) | -0.8 (-1.3, -0.3) | 62.8 (50.6, 74.9) |  | -0.1 (-0.4, 0.2) | -0.1 (-0.3, 0.1) | -0.4 (-0.8, 0.0) | 58.5 (48.2, 68.8) |
| Education |  |  |  |  |  |  |  |  |  |
| College or university | Reference | Reference | Reference | Reference |  | Reference | Reference | Reference | Reference |
| Middle or high school | 0.9 (-0.1, 1.9) | 0.6 (-0.1, 1.2) | 1.4 (0.1, 2.8) | 24.2 (-8.0, 56.3) |  | 0.7 (0.0, 1.3) | 0.6 (0.0, 1.1) | 0.7 (-0.1, 1.6) | -8.7 (-30.8, 13.5) |
| Primary school or below | 1.2 (0.2, 2.3) | 0.8 (0.1, 1.5) | 1.9 (0.5, 3.3) | 33.7 (0.0, 67.4) |  | 0.8 (0.1, 1.4) | 0.6 (0.0, 1.2) | 0.9 (-0.1, 1.8) | -0.2 (-24.0, 23.6) |
| Smoking |  |  |  |  |  |  |  |  |  |
| Never | Reference | Reference | Reference | Reference |  | Reference | Reference | Reference | Reference |
| Former | 0.0 (-0.8, 0.9) | 0.1 (-0.5, 0.6) | 0.1 (-1.0, 1.2) | -13.4 (-39.6, 12.9) |  | -0.3 (-1.0, 0.4) | -0.2 (-0.8, 0.4) | -0.3 (-1.3, 0.7) | -25.6 (-50.9, -0.2) |
| Current, cigarettes (or equivalent)/d | |  |  |  |  |  |  |  |  |
| <20 | 0.6 (-0.1, 1.2) | 0.6 (0.2, 1.1) | 0.6 (-0.2, 1.5) | -37.6 (-58.3, -16.9) |  | -0.2 (-0.7, 0.3) | -0.1 (-0.5, 0.4) | -0.4 (-1.1, 0.2) | -30.1 (-47.1, -13.0) |
| ≥20 | 0.8 (0.1, 1.4) | 0.7 (0.3, 1.1) | 0.7 (-0.2, 1.5) | -30.0 (-50.6, -9.4) |  | 0.0 (-0.5, 0.4) | 0.0 (-0.4, 0.5) | -0.1 (-0.8, 0.6) | -24.1 (-41.2, -7.1) |
| Alcohol drinking |  |  |  |  |  |  |  |  |  |
| Less than weekly | Reference | Reference | Reference | Reference |  | Reference | Reference | Reference | Reference |
| Former | 0.6 (-0.3, 1.4) | 0.2 (-0.3, 0.8) | 0.2 (-0.9, 1.3) | 49.9 (22.7, 77.1) |  | 0.1 (-0.7, 0.8) | 0.1 (-0.6, 0.7) | 0.1 (-0.9, 1.2) | 3.9 (-22.2, 29.9) |
| Weekly | -0.3 (-1.1, 0.4) | -0.4 (-0.9, 0.1) | -0.5 (-1.5, 0.4) | 39.3 (16.3, 62.3) |  | 0.3 (-0.3, 0.8) | 0.2 (-0.3, 0.6) | 0.3 (-0.4, 1.1) | 15.5 (-3.7, 34.8) |
| Daily, g/d (pure alcohol) |  |  |  |  |  |  |  |  |  |
| <30 | 0.4 (-0.7, 1.4) | 0.2 (-0.5, 0.9) | -0.4 (-1.8, 1.0) | 31.9 (-2.4, 66.2) |  | 0.7 (-0.2, 1.6) | 0.6 (-0.2, 1.4) | 0.7 (-0.5, 2.0) | 13.9 (-17.4, 45.3) |
| 30–59 | 0.6 (-0.4, 1.5) | 0.3 (-0.4, 0.9) | 0.4 (-0.8, 1.7) | 37.0 (6.2, 67.9) |  | 1.1 (0.3, 1.9) | 0.9 (0.2, 1.6) | 1.5 (0.4, 2.6) | 41.6 (14.0, 69.3) |
| ≥60 | 0.3 (-0.5, 1.2) | 0.1 (-0.5, 0.6) | 0.4 (-0.7, 1.5) | 55.3 (28.0, 82.6) |  | 0.8 (0.1, 1.6) | 0.6 (-0.0, 1.3) | 1.6 (0.6, 2.7) | 22.7 (-3.9, 49.3) |
| Fresh vegetable consumption |  |  |  |  |  |  |  |  |  |
| Daily | Reference | Reference | Reference | Reference |  | Reference | Reference | Reference | Reference |
| Nondaily | -0.7 (-1.5, 0.1) | -0.6 (-1.1, -0.0) | -1.3 (-2.4, -0.3) | 23.3 (-1.7, 48.3) |  | 0.3 (-0.2, 0.9) | 0.3 (-0.2, 0.8) | 0.4 (-0.4, 1.2) | -1.9 (-21.7, 17.8) |
| Fresh fruit consumption |  |  |  |  |  |  |  |  |  |
| Daily | Reference | Reference | Reference | Reference |  | Reference | Reference | Reference | Reference |
| Nondaily | 0.4 (-0.1, 0.9) | 0.2 (-0.2, 0.5) | 0.4 (-0.2, 1.1) | -4.8 (-21.1, 11.4) |  | -0.2 (-0.5, 0.2) | -0.2 (-0.5, 0.1) | -0.2 (-0.7, 0.3) | -10.9 (-23.6, 1.8) |
| Red meat consumption |  |  |  |  |  |  |  |  |  |
| Daily | Reference | Reference | Reference | Reference |  | Reference | Reference | Reference | Reference |
| Weekly | 0.2 (-0.2, 0.6) | 0.1 (-0.2, 0.3) | 0.4 (-0.2, 0.9) | 5.1 (-8.4, 18.6) |  | -0.1 (-0.4, 0.2) | -0.1 (-0.3, 0.2) | -0.1 (-0.5, 0.3) | -8.3 (-19.0, 2.4) |
| Less than weekly | 0.2 (-0.4, 0.8) | 0.1 (-0.3, 0.5) | 0.4 (-0.4, 1.1) | 36.2 (17.1, 55.4) |  | 0.1 (-0.4, 0.5) | 0.1 (-0.3, 0.5) | -0.1 (-0.7, 0.5) | 5.2 (-10.5, 21.0) |
| Total physical activity,  per 4 MET-h/d^*^ | -0.02 (-0.07, 0.03) | -0.01 (-0.04, 0.02) | 0.02 (-0.05, 0.08) | -1.97 (-3.56, -0.38) |  | -0.03 (-0.06, 0.01) | -0.03 (-0.06, 0.01) | -0.02 (-0.07, 0.03) | -1.45 (-2.69, -0.20) |
| BMI, per 5 kg/m^2^ | -0.6 (-0.8, -0.3) | -0.8 (-0.9, -0.6) | -0.3 (-0.6, 0.0) | 73.7 (65.8, 81.5) |  | -0.2 (-0.4, -0.0) | -0.3 (-0.5, -0.2) | -0.2 (-0.5, 0.1) | 40.5 (33.4, 47.5) |
| WC, per 10 cm | -0.4 (-0.6, -0.3) | -0.5 (-0.7, -0.4) | -0.2 (-0.5, -0.0) | 32.2 (26.5, 37.8) |  | -0.2 (-0.3, -0.0) | -0.2 (-0.3, -0.1) | -0.1 (-0.3, 0.1) | 15.9 (10.9, 21.0) |
| Body shape |  |  |  |  |  |  |  |  |  |
| BMI<18.5 | 1.4 (0.4, 2.4) | 1.4 (0.7, 2.1) | 1.4 (0.1, 2.7) | -73.1 (-105.4, -40.8) |  | 0.0 (-0.5, 0.5) | 0.1 (-0.3, 0.5) | -0.1 (-0.8, 0.6) | -36.8 (-53.9, -19.6) |
| BMI 18.5–23.9, WC<90/85 | Reference | Reference | Reference | Reference |  | Reference | Reference | Reference | Reference |
| BMI 18.5–23.9, WC≥90/85 | -1.4 (-2.9, 0.1) | -1.2 (-2.2, -0.1) | -0.8 (-2.8, 1.1) | 35.7 (-12.5, 84.0) |  | -0.4 (-1.7, 1.0) | -0.3 (-1.5, 0.9) | -0.1 (-2.0, 1.8) | -14.9 (-62.7, 33.0) |
| BMI 24.0–27.9, WC<90/85 | -0.2 (-0.6, 0.2) | -0.4 (-0.7, -0.2) | 0.3 (-0.2, 0.8) | 65.7 (53.4, 78.1) |  | -0.3 (-0.6, -0.0) | -0.4 (-0.6, -0.1) | -0.2 (-0.6, 0.2) | 38.5 (27.9, 49.1) |
| BMI 24.0–27.9, WC≥90/85 | -1.0 (-1.5, -0.5) | -1.0 (-1.3, -0.7) | -0.9 (-1.6, -0.3) | 38.7 (22.5, 54.9) |  | -0.3 (-0.8, 0.2) | -0.4 (-0.8, 0.1) | -0.4 (-1.2, 0.3) | 18.5 (0.3, 36.7) |
| BMI≥28.0, WC<90/85 | -0.1 (-1.4, 1.1) | -0.5 (-1.4, 0.3) | 0.0 (-1.6, 1.7) | 104.9 (64.9, 144.8) |  | -0.1 (-1.3, 1.2) | -0.3 (-1.3, 0.8) | -0.6 (-2.3, 1.1) | 60.9 (17.5, 104.3) |
| BMI≥28.0, WC≥90/85 | -1.0 (-1.5, -0.5) | -1.2 (-1.6, -0.9) | -0.4 (-1.1, 0.3) | 112.4 (95.3, 129.6) |  | -0.3 (-0.9, 0.3) | -0.5 (-1.0, 0.0) | 0.0 (-0.9, 0.8) | 53.8 (32.3, 75.3) |

The table presents the adjusted difference (95% confidence interval) in SBP change indices for categorical variables compared with the reference group and adjusted index change (95% CI) per specified unit for continuous variables in hypertension and non-hypertension participants. Please refer to the method section for detailed covariate adjustment.

*The 4 MET-h/d is equivalent to about 1 hour of moderate physical activity per day.

ARV, average real variability (mmHg); BMI, body mass index; cumSBP, cumulative systolic blood pressure (100 mmHg×year); CV, coefficient of variation (%); MET-h/d, metabolic equivalent task-hour/day; SD, standard deviation (mmHg); WC, waist circumference.

**Table S5.** The adjusted differences in DBP change indices by baseline characteristics in hypertensive and normotensive participants

|  | Hypertension (n = 10,751) | | | |  | Normotension (n = 5234) | | | |
| --- | --- | --- | --- | --- | --- | --- | --- | --- | --- |
|  | SD | CV | ARV | cumDBP |  | SD | CV | ARV | cumDBP |
| Mean values | 7.9 | 9.3 | 9.5 | 1298.0 |  | 5.5 | 7.7 | 6.8 | 1100.4 |
| Age, per 10 years | -0.5 (-0.7, -0.4) | -0.5 (-0.6, -0.3) | -0.4 (-0.6, -0.3) | -30.5 (-34.3, -26.8) |  | -0.1 (-0.2, 0.0) | 0.1 (-0.1, 0.2) | 0.0 (-0.2, 0.1) | -22.6 (-26.5, -18.7) |
| Sex |  |  |  |  |  |  |  |  |  |
| Women | Reference | Reference | Reference | Reference |  | Reference | Reference | Reference | Reference |
| Men | 0.4 (0.1, 0.7) | 0.3 (-0.1, 0.6) | 0.5 (0.1, 0.8) | 27.0 (16.8, 37.1) |  | 0.1 (-0.2, 0.4) | -0.1 (-0.5, 0.4) | 0.1 (-0.3, 0.5) | 25.9 (15.6, 36.1) |
| Residence |  |  |  |  |  |  |  |  |  |
| Urban | Reference | Reference | Reference | Reference |  | Reference | Reference | Reference | Reference |
| Rural | 0.2 (-0.0, 0.4) | 0.2 (-0.1, 0.4) | 0.2 (-0.1, 0.5) | 43.0 (36.0, 50.0) |  | 0.1 (-0.1, 0.3) | 0.2 (-0.1, 0.4) | 0.1 (-0.2, 0.4) | 38.0 (30.9, 45.1) |
| Education |  |  |  |  |  |  |  |  |  |
| College or university | Reference | Reference | Reference | Reference |  | Reference | Reference | Reference | Reference |
| Middle or high school | 0.3 (-0.3, 0.8) | 0.3 (-0.3, 0.9) | 0.5 (-0.2, 1.2) | -0.4 (-19.0, 18.1) |  | 0.6 (0.1, 1.0) | 0.8 (0.1, 1.4) | 0.3 (-0.2, 0.9) | -3.3 (-18.7, 12.1) |
| Primary school or below | 0.5 (-0.1, 1.1) | 0.6 (-0.0, 1.3) | 0.8 (0.0, 1.5) | 0.5 (-19.0, 20.0) |  | 0.5 (0.0, 1.0) | 0.7 (0.0, 1.4) | 0.4 (-0.3, 1.0) | -2.6 (-19.1, 14.0) |
| Smoking |  |  |  |  |  |  |  |  |  |
| Never | Reference | Reference | Reference | Reference |  | Reference | Reference | Reference | Reference |
| Former | 0.1 (-0.4, 0.5) | 0.1 (-0.4, 0.6) | 0.0 (-0.5, 0.6) | -6.3 (-21.5, 8.9) |  | -0.5 (-1.0, 0.0) | -0.6 (-1.4, 0.1) | -0.3 (-1.0, 0.3) | -12.6 (-30.2, 5.1) |
| Current, cigarettes (or equivalent)/d | |  |  |  |  |  |  |  |  |
| <20 | 0.0 (-0.4, 0.3) | 0.2 (-0.2, 0.6) | -0.1 (-0.5, 0.4) | -29.1 (-41.1, -17.2) |  | 0.1 (-0.3, 0.4) | 0.2 (-0.3, 0.7) | 0.1 (-0.4, 0.5) | -14.0 (-25.9, -2.1) |
| ≥20 | 0.1 (-0.3, 0.4) | 0.3 (-0.1, 0.7) | 0.0 (-0.5, 0.4) | -25.5 (-37.4, -13.6) |  | 0.1 (-0.2, 0.5) | 0.2 (-0.3, 0.7) | 0.1 (-0.4, 0.6) | -7.3 (-19.1, 4.6) |
| Alcohol drinking |  |  |  |  |  |  |  |  |  |
| Less than weekly | Reference | Reference | Reference | Reference |  | Reference | Reference | Reference | Reference |
| Former | 0.4 (-0.1, 0.8) | 0.3 (-0.2, 0.9) | 0.3 (-0.2, 0.9) | 28.5 (12.7, 44.2) |  | 0.1 (-0.4, 0.7) | 0.1 (-0.6, 0.9) | 0.0 (-0.7, 0.7) | 10.6 (-7.5, 28.7) |
| Weekly | 0.3 (-0.1, 0.7) | 0.2 (-0.3, 0.6) | 0.4 (-0.1, 0.9) | 24.7 (11.3, 38.0) |  | 0.6 (0.2, 1.0) | 0.8 (0.2, 1.3) | 0.6 (0.1, 1.1) | 14.1 (0.7, 27.5) |
| Daily, g/d (pure alcohol) |  |  |  |  |  |  |  |  |  |
| <30 | 0.2 (-0.4, 0.7) | 0.1 (-0.5, 0.8) | -0.1 (-0.9, 0.6) | 32.9 (13.0, 52.7) |  | 0.1 (-0.6, 0.7) | 0.0 (-0.9, 0.9) | 0.3 (-0.6, 1.1) | 16.9 (-5.0, 38.7) |
| 30–59 | 0.5 (0.0, 1.0) | 0.5 (-0.1, 1.1) | 0.7 (0.0, 1.4) | 29.7 (11.8, 47.5) |  | 0.4 (-0.2, 0.9) | 0.4 (-0.4, 1.2) | 0.5 (-0.3, 1.2) | 30.7 (11.4, 49.9) |
| ≥60 | 0.3 (-0.2, 0.8) | 0.2 (-0.3, 0.8) | 0.5 (-0.1, 1.1) | 33.6 (17.8, 49.4) |  | 1.0 (0.4, 1.5) | 1.2 (0.4, 1.9) | 1.2 (0.5, 1.9) | 25.4 (6.9, 43.9) |
| Fresh vegetable consumption |  |  |  |  |  |  |  |  |  |
| Daily | Reference | Reference | Reference | Reference |  | Reference | Reference | Reference | Reference |
| Nondaily | -0.3 (-0.7, 0.1) | -0.4 (-0.9, 0.1) | -0.4 (-1.0, 0.1) | 9.4 (-5.1, 23.8) |  | -0.3 (-0.6, 0.1) | -0.4 (-1.0, 0.1) | -0.1 (-0.7, 0.4) | 5.4 (-8.3, 19.1) |
| Fresh fruit consumption |  |  |  |  |  |  |  |  |  |
| Daily | Reference | Reference | Reference | Reference |  | Reference | Reference | Reference | Reference |
| Nondaily | 0.0 (-0.2, 0.3) | 0.0 (-0.3, 0.3) | 0.1 (-0.3, 0.5) | -5.8 (-15.2, 3.6) |  | 0.0 (-0.3, 0.2) | 0.0 (-0.4, 0.4) | 0.0 (-0.3, 0.4) | -10.6 (-19.5, -1.8) |
| Red meat consumption |  |  |  |  |  |  |  |  |  |
| Daily | Reference | Reference | Reference | Reference |  | Reference | Reference | Reference | Reference |
| Weekly | 0.1 (-0.1, 0.3) | 0.1 (-0.2, 0.4) | 0.2 (-0.1, 0.5) | 0.1 (-7.7, 7.9) |  | 0.0 (-0.2, 0.2) | 0.0 (-0.3, 0.3) | 0.0 (-0.3, 0.3) | -2.7 (-10.2, 4.7) |
| Less than weekly | 0.3 (-0.0, 0.6) | 0.4 (-0.0, 0.7) | 0.3 (-0.1, 0.7) | 8.6 (-2.4, 19.7) |  | 0.1 (-0.2, 0.4) | 0.1 (-0.3, 0.6) | -0.1 (-0.6, 0.3) | 10.3 (-0.6, 21.3) |
| Total physical activity,  per 4 MET-h/d^*^ | 0.00 (-0.03, 0.02) | 0.00 (-0.03, 0.03) | 0.00 (-0.03, 0.04) | -1.27 (-2.19, -0.36) |  | 0.00 (-0.02, 0.03) | 0.00 (-0.03, 0.04) | 0.01 (-0.02, 0.04) | -1.21 (-2.07, -0.34) |
| BMI, per 5 kg/m^2^ | -0.1 (-0.3, -0.0) | -0.5 (-0.6, -0.3) | 0.1 (-0.1, 0.2) | 47.4 (42.9, 52.0) |  | -0.1 (-0.2, 0.1) | -0.3 (-0.5, -0.1) | -0.1 (-0.3, 0.1) | 31.9 (26.9, 36.8) |
| WC, per 10 cm | -0.1 (-0.2, -0.0) | -0.3 (-0.4, -0.2) | 0.0 (-0.1, 0.1) | 22.2 (19.0, 25.5) |  | -0.1 (-0.2, 0.0) | -0.2 (-0.4, -0.1) | -0.1 (-0.2, 0.0) | 15.4 (11.8, 18.9) |
| Body shape |  |  |  |  |  |  |  |  |  |
| BMI<18.5 | 0.2 (-0.4, 0.7) | 0.4 (-0.2, 1.0) | 0.2 (-0.5, 0.9) | -33.6 (-52.2, -14.9) |  | 0.0 (-0.3, 0.4) | 0.2 (-0.3, 0.7) | 0.0 (-0.4, 0.5) | -26.0 (-37.9, -14.1) |
| BMI 18.5–23.9, WC<90/85 | Reference | Reference | Reference | Reference |  | Reference | Reference | Reference | Reference |
| BMI 18.5–23.9, WC≥90/85 | -0.8 (-1.6, 0.0) | -1.2 (-2.1, -0.3) | -0.9 (-2.0, 0.1) | 25.4 (-2.5, 53.3) |  | -0.2 (-1.2, 0.8) | -0.3 (-1.7, 1.1) | -0.1 (-1.3, 1.2) | -12.1 (-45.3, 21.2) |
| BMI 24.0–27.9, WC<90/85 | -0.2 (-0.4, -0.0) | -0.5 (-0.8, -0.3) | -0.1 (-0.3, 0.2) | 43.6 (36.5, 50.8) |  | -0.1 (-0.3, 0.2) | -0.3 (-0.6, 0.1) | 0.1 (-0.2, 0.4) | 30.9 (23.5, 38.2) |
| BMI 24.0–27.9, WC≥90/85 | -0.3 (-0.6, -0.0) | -0.7 (-1.0, -0.4) | -0.2 (-0.6, 0.1) | 31.7 (22.4, 41.1) |  | -0.3 (-0.6, 0.1) | -0.6 (-1.1, -0.1) | -0.3 (-0.8, 0.2) | 25.1 (12.4, 37.7) |
| BMI≥28.0, WC<90/85 | -0.2 (-0.8, 0.5) | -0.7 (-1.4, 0.1) | -0.1 (-0.9, 0.8) | 81.8 (58.6, 104.9) |  | -0.7 (-1.6, 0.2) | -1.2 (-2.4, 0.1) | -1.0 (-2.2, 0.1) | 60.8 (30.7, 90.9) |
| BMI≥28.0, WC≥90/85 | -0.1 (-0.4, 0.1) | -0.7 (-1.0, -0.3) | 0.3 (-0.1, 0.7) | 71.2 (61.3, 81.1) |  | 0.1 (-0.3, 0.5) | -0.2 (-0.8, 0.5) | 0.1 (-0.4, 0.7) | 44.5 (29.6, 59.4) |

The table presents the adjusted difference (95% confidence interval) in DBP change indices for categorical variables compared with the reference group and adjusted index change (95% CI) per specified unit for continuous variables in hypertension and non-hypertension participants. Please refer to the method section for detailed covariate adjustment.

*The 4 MET-h/d is equivalent to about 1 hour of moderate physical activity per day.

ARV, average real variability (mmHg); BMI, body mass index; cumDBP, cumulative diastolic blood pressure (100 mmHg×year); CV, coefficient of variation (%); MET-h/d, metabolic equivalent task-hour/day; SD, standard deviation (mmHg); WC, waist circumference.

**Table S6.** The adjusted differences in SBP and DBP change indices by baseline characteristics in participants without antihypertensive treatment

|  | SBP | | | |  | DBP | | | |
| --- | --- | --- | --- | --- | --- | --- | --- | --- | --- |
|  | SD | CV | ARV | cumSBP |  | SD | CV | ARV | cumDBP |
| Mean values | 11.1 | 8.7 | 13.4 | 1925.5 |  | 6.3 | 8.3 | 7.6 | 1152.0 |
| Age, per 10 years | 1.4 (1.2, 1.6) | 0.8 (0.7, 0.9) | 1.7 (1.5, 2.0) | 65.7 (59.8, 71.6) |  | -0.2 (-0.2, -0.1) | -0.1 (-0.2, 0.1) | -0.1 (-0.2, 0.1) | -17.2 (-20.7, -13.8) |
| Sex |  |  |  |  |  |  |  |  |  |
| Women | Reference | Reference | Reference | Reference |  | Reference | Reference | Reference | Reference |
| Men | -0.9 (-1.4, -0.4) | -0.8 (-1.1, -0.4) | -1.0 (-1.6, -0.4) | 22.6 (6.5, 38.7) |  | 0.3 (0.0, 0.5) | 0.2 (-0.2, 0.5) | 0.3 (0.0, 0.7) | 29.3 (19.9, 38.7) |
| Residence |  |  |  |  |  |  |  |  |  |
| Urban | Reference | Reference | Reference | Reference |  | Reference | Reference | Reference | Reference |
| Rural | 0.1 (-0.2, 0.5) | 0.1 (-0.2, 0.3) | -0.4 (-0.9, -0.0) | 55.5 (44.2, 66.8) |  | 0.1 (-0.1, 0.3) | 0.1 (-0.1, 0.4) | 0.1 (-0.1, 0.4) | 39.1 (32.5, 45.7) |
| Education |  |  |  |  |  |  |  |  |  |
| College or university | Reference | Reference | Reference | Reference |  | Reference | Reference | Reference | Reference |
| Middle or high school | 1.5 (0.8, 2.3) | 1.1 (0.5, 1.6) | 1.8 (0.8, 2.8) | 32.8 (7.0, 58.7) |  | 0.8 (0.3, 1.2) | 0.9 (0.4, 1.5) | 0.8 (0.2, 1.3) | 11.6 (-3.6, 26.7) |
| Primary school or below | 1.8 (1.0, 2.6) | 1.3 (0.7, 1.9) | 2.1 (1.1, 3.2) | 36.9 (9.3, 64.5) |  | 0.8 (0.4, 1.3) | 1.0 (0.4, 1.6) | 0.9 (0.3, 1.5) | 10.8 (-5.4, 26.9) |
| Smoking |  |  |  |  |  |  |  |  |  |
| Never | Reference | Reference | Reference | Reference |  | Reference | Reference | Reference | Reference |
| Former | 0.1 (-0.7, 0.9) | 0.1 (-0.5, 0.7) | 0.2 (-0.8, 1.2) | -29.5 (-56.2, -2.8) |  | -0.1 (-0.6, 0.3) | -0.2 (-0.8, 0.4) | 0.0 (-0.5, 0.6) | -10.4 (-26.0, 5.2) |
| Current, cigarettes (or equivalent)/d | |  |  |  |  |  |  |  |  |
| <20 | 0.5 (-0.1, 1.0) | 0.4 (0.0, 0.9) | 0.4 (-0.3, 1.1) | -21.9 (-40.4, -3.4) |  | 0.3 (-0.0, 0.6) | 0.5 (0.1, 0.9) | 0.3 (-0.1, 0.7) | -10.6 (-21.4, 0.3) |
| ≥20 | 0.2 (-0.4, 0.7) | 0.3 (-0.2, 0.7) | 0.1 (-0.6, 0.8) | -31.9 (-50.7, -13.1) |  | 0.1 (-0.2, 0.4) | 0.2 (-0.2, 0.6) | 0.0 (-0.4, 0.4) | -16.8 (-27.8, -5.9) |
| Alcohol drinking |  |  |  |  |  |  |  |  |  |
| Less than weekly | Reference | Reference | Reference | Reference |  | Reference | Reference | Reference | Reference |
| Former | 0.1 (-0.8, 0.9) | 0.1 (-0.6, 0.7) | -0.3 (-1.4, 0.7) | 3.7 (-24.5, 32.0) |  | 0.4 (-0.1, 0.8) | 0.5 (-0.1, 1.1) | 0.4 (-0.2, 1.0) | 13.1 (-3.4, 29.6) |
| Weekly | 0.3 (-0.4, 0.9) | 0.1 (-0.4, 0.6) | 0.0 (-0.8, 0.8) | 39.4 (18.6, 60.2) |  | 0.5 (0.2, 0.9) | 0.6 (0.1, 1.0) | 0.5 (0.1, 1.0) | 25.3 (13.1, 37.4) |
| Daily, g/d (pure alcohol) |  |  |  |  |  |  |  |  |  |
| <30 | 0.9 (-0.1, 1.9) | 0.6 (-0.1, 1.4) | 0.6 (-0.7, 1.8) | 33.9 (0.8, 67.0) |  | 0.0 (-0.5, 0.6) | -0.1 (-0.8, 0.6) | -0.1 (-0.8, 0.6) | 33.5 (14.1, 52.8) |
| 30–59 | 0.9 (0.1, 1.8) | 0.6 (-0.0, 1.3) | 1.1 (0.0, 2.2) | 49.3 (20.9, 77.7) |  | 0.5 (0.0, 0.9) | 0.4 (-0.2, 1.0) | 0.5 (-0.1, 1.1) | 36.6 (20.0, 53.2) |
| ≥60 | 1.6 (0.8, 2.4) | 1.0 (0.4, 1.5) | 1.9 (1.0, 2.9) | 80.6 (54.6, 106.5) |  | 1.1 (0.6, 1.5) | 1.1 (0.5, 1.6) | 1.3 (0.8, 1.8) | 54.2 (39.1, 69.4) |
| Fresh vegetable consumption |  |  |  |  |  |  |  |  |  |
| Daily | Reference | Reference | Reference | Reference |  | Reference | Reference | Reference | Reference |
| Nondaily | 0.2 (-0.5, 0.9) | 0.1 (-0.4, 0.6) | 0.1 (-0.8, 0.9) | 16.2 (-5.9, 38.3) |  | -0.4 (-0.7, -0.0) | -0.5 (-1.0, -0.0) | -0.3 (-0.7, 0.2) | 9.1 (-3.8, 22.0) |
| Fresh fruit consumption |  |  |  |  |  |  |  |  |  |
| Daily | Reference | Reference | Reference | Reference |  | Reference | Reference | Reference | Reference |
| Nondaily | -0.1 (-0.6, 0.3) | -0.2 (-0.5, 0.2) | 0.0 (-0.5, 0.6) | -8.0 (-22.3, 6.3) |  | -0.0 (-0.2, 0.2) | -0.0 (-0.3, 0.3) | 0.1 (-0.2, 0.4) | -9.1 (-17.5, -0.7) |
| Red meat consumption |  |  |  |  |  |  |  |  |  |
| Daily | Reference | Reference | Reference | Reference |  | Reference | Reference | Reference | Reference |
| Weekly | -0.4 (-0.7, 0.0) | -0.3 (-0.5, 0.0) | -0.3 (-0.8, 0.1) | -5.8 (-17.8, 6.1) |  | -0.1 (-0.3, 0.1) | -0.1 (-0.4, 0.2) | -0.1 (-0.3, 0.2) | -1.7 (-8.7, 5.3) |
| Less than weekly | 0.1 (-0.5, 0.6) | 0.1 (-0.3, 0.5) | -0.0 (-0.7, 0.6) | 10.5 (-7.0, 28.1) |  | 0.2 (-0.1, 0.5) | 0.3 (-0.1, 0.6) | 0.0 (-0.3, 0.4) | 10.4 (0.1, 20.6) |
| Total physical activity,  per 4 MET-h/d^*^ | -0.03 (-0.07, 0.01) | -0.02 (-0.05, 0.01) | -0.02 (-0.07, 0.04) | -2.28 (-3.67, -0.89) |  | -0.00 (-0.03, 0.02) | 0.00 (-0.03, 0.03) | -0.00 (-0.03, 0.03) | -1.75 (-2.57, -0.94) |
| BMI, per 5 kg/m^2^ | 0.1 (-0.1, 0.4) | -0.2 (-0.3, 0.0) | 0.2 (-0.1, 0.5) | 66.3 (58.6, 73.9) |  | -0.0 (-0.1, 0.1) | -0.3 (-0.4, -0.1) | 0.0 (-0.1, 0.2) | 43.6 (39.1, 48.1) |
| WC, per 10 cm | 0.1 (-0.1, 0.2) | -0.1 (-0.2, 0.0) | 0.1 (-0.1, 0.3) | 30.1 (24.6, 35.5) |  | -0.0 (-0.1, 0.0) | -0.2 (-0.4, -0.1) | -0.1 (-0.2, 0.1) | 21.6 (18.4, 24.8) |
| Body shape |  |  |  |  |  |  |  |  |  |
| BMI<18.5 | -0.0 (-0.7, 0.6) | 0.2 (-0.2, 0.7) | 0.0 (-0.8, 0.8) | -67.9 (-88.6, -47.2) |  | -0.1 (-0.4, 0.3) | 0.1 (-0.4, 0.5) | -0.1 (-0.6, 0.3) | -32.7 (-44.8, -20.6) |
| BMI 18.5–23.9, WC<90/85 | Reference | Reference | Reference | Reference |  | Reference | Reference | Reference | Reference |
| BMI 18.5–23.9, WC≥90/85 | -0.7 (-2.3, 0.8) | -0.7 (-1.8, 0.5) | 0.6 (-1.3, 2.5) | 18.5 (-32.2, 69.2) |  | -0.4 (-1.3, 0.4) | -0.8 (-1.9, 0.3) | -0.3 (-1.4, 0.8) | 16.5 (-13.1, 46.1) |
| BMI 24.0–27.9, WC<90/85 | 0.2 (-0.1, 0.5) | -0.1 (-0.3, 0.2) | 0.4 (-0.0, 0.8) | 63.2 (51.7, 74.6) |  | -0.0 (-0.2, 0.2) | -0.3 (-0.5, -0.0) | 0.0 (-0.2, 0.3) | 41.5 (34.8, 48.2) |
| BMI 24.0–27.9, WC≥90/85 | 0.0 (-0.5, 0.6) | -0.2 (-0.6, 0.2) | -0.1 (-0.8, 0.6) | 48.3 (30.1, 66.4) |  | -0.2 (-0.5, 0.1) | -0.6 (-1.0, -0.2) | -0.3 (-0.7, 0.1) | 36.2 (25.6, 46.8) |
| BMI≥28.0, WC<90/85 | 1.1 (-0.3, 2.5) | 0.7 (-0.4, 1.7) | 1.5 (-0.3, 3.3) | 62.6 (15.7, 109.4) |  | 0.1 (-0.6, 0.9) | -0.1 (-1.1, 0.9) | 0.2 (-0.7, 1.2) | 64.6 (37.2, 92.0) |
| BMI≥28.0, WC≥90/85 | 0.3 (-0.4, 0.9) | -0.2 (-0.7, 0.3) | 0.4 (-0.4, 1.2) | 93.5 (72.4, 114.7) |  | 0.2 (-0.2, 0.5) | -0.2 (-0.6, 0.3) | 0.3 (-0.1, 0.8) | 66.3 (53.9, 78.6) |

The table presents the adjusted difference (95% confidence interval) in SBP and DBP change indices for categorical variables compared with the reference group and adjusted SBP/DBP indices change (95% CI) per specified unit for continuous variables in participants without antihypertensive treatment. Please refer to the method section for detailed covariate adjustment.

*The 4 MET-h/d is equivalent to about 1 hour of moderate physical activity per day.

ARV, average real variability (mmHg); BMI, body mass index; cumDBP, cumulative diastolic blood pressure (100 mmHg×year); cumSBP, cumulative systolic blood pressure (100 mmHg×year); CV, coefficient of variation (%); MET-h/d, metabolic equivalent task-hour/day; SD, standard deviation (mmHg); WC, waist circumference.

**Table S7.** The baseline characteristics of participants with hypertension, normotension, and without anti-hypertensive treatment

|  | Hypertension | Normotension | Without anti-hypertensive treatment |
| --- | --- | --- | --- |
| Number | 10,751 | 5,234 | 8,978 |
| Age, years | 52.0 | 46.6 | 48.1 |
| Men, % | 37.2 | 33.7 | 35.8 |
| Rural, % | 67.2 | 69.2 | 69.3 |
| Primary school or below, % | 60.2 | 50.2 | 53.0 |
| Smoking daily, % | 24.5 | 23.6 | 24.9 |
| Drinking daily, % | 9.2 | 6.7 | 8.3 |
| Daily food consumption, % |  |  |  |
| Fresh fruits | 13.3 | 14.5 | 13.8 |
| Fresh vegetables | 95.6 | 95.3 | 95.4 |
| Red meat | 24.1 | 25.3 | 25.1 |
| Total physical activity, MET-h/d | 22.4 | 25.2 | 24.3 |
| BMI, kg/m^2^ | 24.2 | 22.5 | 22.9 |
| WC, cm | 80.4 | 75.7 | 76.7 |
| SBP, mmHg | 137.6 | 115.9 | 121.6 |
| DBP, mmHg | 81.2 | 70.8 | 73.7 |

BMI, body mass index; DBP, diastolic blood pressure; MET-h/d, metabolic equivalent task-hour/day; SBP, systolic blood pressure; WC, waist circumference.

**Table S8.** Association between baseline characteristics and DBP trajectory groups

|  | G2 | G3 | G4 | G5 |
| --- | --- | --- | --- | --- |
| Age, years |  |  |  |  |
| <50 | Reference | Reference | Reference | Reference |
| 50–59 | 0.94 (0.84, 1.06) | 0.97 (0.86, 1.10) | 0.66 (0.52, 0.84) | 1.54 (1.27, 1.87) |
| ≥60 | 0.93 (0.80, 1.08) | 0.70 (0.59, 0.83) | 0.58 (0.41, 0.81) | 1.19 (0.92, 1.53) |
| Sex |  |  |  |  |
| Women | Reference | Reference | Reference | Reference |
| Men | 1.27 (1.08, 1.50) | 1.33 (1.10, 1.60) | 1.63 (1.16, 2.30) | 1.81 (1.38, 2.39) |
| Residence |  |  |  |  |
| Urban | Reference | Reference | Reference | Reference |
| Rural | 0.99 (0.89, 1.11) | 1.01 (0.89, 1.14) | 1.08 (0.84, 1.37) | 0.93 (0.77, 1.12) |
| Education |  |  |  |  |
| College or university | Reference | Reference | Reference | Reference |
| Middle or high school | 1.24 (0.95, 1.61) | 1.15 (0.85, 1.56) | 1.58 (0.86, 2.90) | 1.34 (0.83, 2.18) |
| Primary school or below | 1.09 (0.82, 1.45) | 1.06 (0.77, 1.46) | 1.20 (0.63, 2.30) | 1.36 (0.81, 2.26) |
| Smoking |  |  |  |  |
| Never | Reference | Reference | Reference | Reference |
| Former | 0.88 (0.67, 1.17) | 1.11 (0.82, 1.49) | 0.86 (0.51, 1.46) | 0.80 (0.53, 1.21) |
| Current, cigarettes (or equivalent)/d | |  |  |  |
| <20 | 0.78 (0.65, 0.95) | 0.79 (0.64, 0.98) | 0.49 (0.32, 0.75) | 0.58 (0.42, 0.81) |
| ≥20 | 0.80 (0.66, 0.98) | 0.80 (0.64, 1.00) | 0.67 (0.45, 0.99) | 0.63 (0.46, 0.87) |
| Alcohol drinking |  |  |  |  |
| Less than weekly | Reference | Reference | Reference | Reference |
| Former | 1.26 (0.96, 1.67) | 1.51 (1.11, 2.05) | 2.12 (1.24, 3.61) | 2.12 (1.41, 3.18) |
| Weekly | 1.39 (1.10, 1.77) | 1.67 (1.29, 2.16) | 2.20 (1.44, 3.34) | 1.82 (1.26, 2.64) |
| Daily, g/d (pure alcohol) |  |  |  |  |
| <30 | 1.15 (0.83, 1.61) | 1.39 (0.96, 2.01) | 1.70 (0.85, 3.41) | 1.71 (1.01, 2.89) |
| 30–59 | 1.27 (0.94, 1.71) | 1.48 (1.06, 2.06) | 1.67 (0.87, 3.18) | 1.77 (1.09, 2.85) |
| ≥60 | 2.40 (1.77, 3.26) | 2.64 (1.88, 3.69) | 2.90 (1.58, 5.32) | 3.35 (2.11, 5.32) |
| Fresh vegetable consumption |  |  |  |  |
| Daily | Reference | Reference | Reference | Reference |
| Nondaily | 1.19 (0.94, 1.51) | 1.14 (0.88, 1.49) | 1.64 (1.05, 2.54) | 1.14 (0.75, 1.72) |
| Fresh fruit consumption |  |  |  |  |
| Daily | Reference | Reference | Reference | Reference |
| Nondaily | 0.91 (0.78, 1.05) | 1.01 (0.86, 1.20) | 1.15 (0.82, 1.61) | 1.21 (0.93, 1.57) |
| Red meat consumption |  |  |  |  |
| Daily | Reference | Reference | Reference | Reference |
| Weekly | 1.05 (0.93, 1.19) | 1.01 (0.88, 1.17) | 0.98 (0.75, 1.29) | 1.07 (0.87, 1.33) |
| Less than weekly | 1.10 (0.92, 1.32) | 0.95 (0.77, 1.16) | 1.23 (0.85, 1.77) | 1.10 (0.82, 1.50) |
| Total physical activity^*^ |  |  |  |  |
| Low | Reference | Reference | Reference | Reference |
| Medium | 1.00 (0.90, 1.12) | 0.96 (0.84, 1.09) | 0.76 (0.59, 0.98) | 0.92 (0.75, 1.11) |
| High | 0.96 (0.85, 1.08) | 0.94 (0.82, 1.08) | 0.78 (0.60, 1.02) | 0.82 (0.67, 1.02) |
| BMI |  |  |  |  |
| <18.5 kg/m^2^ | Reference | Reference | Reference | Reference |
| 18.5–23.9 kg/m^2^ | 1.74 (1.44, 2.11) | 3.01 (2.27, 4.00) | 4.42 (1.79, 10.91) | 2.65 (1.54, 4.55) |
| 24.0–27.9 kg/m^2^ | 3.30 (2.68, 4.06) | 9.64 (7.18, 12.94) | 17.56 (7.09, 43.50) | 11.97 (6.92, 20.71) |
| ≥28.0 kg/m^2^ | 5.19 (3.87, 6.95) | 22.97 (16.04, 32.89) | 40.42 (15.68, 104.22) | 35.13 (19.39, 63.65) |
| WC (men/women) |  |  |  |  |
| <85/80 cm | Reference | Reference | Reference | Reference |
| 85–90/80–85 cm | 1.62 (1.42, 1.86) | 2.76 (2.38, 3.19) | 3.00 (2.29, 3.93) | 3.54 (2.87, 4.36) |
| ≥90/85 cm | 2.32 (1.99, 2.70) | 4.63 (3.95, 5.43) | 6.57 (5.09, 8.47) | 6.43 (5.20, 7.94) |
| Body shape |  |  |  |  |
| BMI<18.5 | 0.58 (0.48, 0.70) | 0.34 (0.25, 0.45) | 0.24 (0.10, 0.59) | 0.38 (0.22, 0.66) |
| BMI 18.5–23.9, WC<90/85 | Reference | Reference | Reference | Reference |
| BMI 18.5–23.9, WC≥90/85 | 1.32 (0.83, 2.11) | 1.66 (0.98, 2.81) | 4.92 (2.33, 10.38) | 1.64 (0.70, 3.88) |
| BMI 24.0–27.9, WC<90/85 | 1.80 (1.59, 2.03) | 2.99 (2.61, 3.41) | 3.62 (2.82, 4.66) | 4.23 (3.46, 5.17) |
| BMI 24.0–27.9, WC≥90/85 | 2.25 (1.86, 2.73) | 4.10 (3.35, 5.03) | 6.13 (4.42, 8.51) | 5.73 (4.35, 7.54) |
| BMI≥28.0, WC<90/85 | 2.82 (1.60, 4.99) | 6.43 (3.61, 11.46) | 3.98 (1.40, 11.31) | 11.49 (5.81, 22.72) |
| BMI≥28.0, WC≥90/85 | 3.03 (2.36, 3.89) | 7.93 (6.15, 10.24) | 10.63 (7.35, 15.37) | 13.76 (10.10, 18.75) |

The table presents the adjusted RRR (95% CI), with G1 being the reference group. Please refer to the method section for detailed covariate adjustment.

^*^Total physical activity level was categorized based on age- (<50, 50–59, and ≥60 years) and sex-specific tertile cutoff points.

G1: optimal, low-growth (reference); G2: normal, low-growth; G3: elevated, low-growth; G4: high DBP, high-growth; G5: extreme high DBP, decrease.

BMI, body mass index; DBP, diastolic blood pressure; RRR, relative risk ratio; WC, waist circumference.

**Table S9.** Association between baseline characteristics and combined blood pressure trajectories

|  | Isolated SBP high-risk trajectory | Isolated DBP high-risk trajectory | Both SBP and DBP high-risk trajectories |  | Isolated DBP high-risk trajectory | Both SBP and DBP high-risk trajectories |  | Both SBP and DBP high-risk trajectories |
| --- | --- | --- | --- | --- | --- | --- | --- | --- |
|  | VS.  Both SBP and DBP low-risk trajectories | | |  | VS.  Isolated SBP high-risk trajectory | |  | VS.  Isolated DBP high-risk trajectory |
| Age, years |  |  |  |  |  |  |  |  |
| <50 | Reference | Reference | Reference |  | Reference | Reference |  | Reference |
| 50–59 | 1.24 (1.11, 1.39) | 2.47 (1.72, 3.57) | 1.13 (0.98, 1.31) |  | 1.99 (1.37, 2.90) | 0.91 (0.77, 1.08) |  | 0.46 (0.31, 0.67) |
| ≥60 | 1.44 (1.24, 1.67) | 3.69 (2.42, 5.62) | 0.90 (0.73, 1.11) |  | 2.56 (1.66, 3.96) | 0.62 (0.49, 0.79) |  | 0.24 (0.15, 0.39) |
| Sex |  |  |  |  |  |  |  |  |
| Women | Reference | Reference | Reference |  | Reference | Reference |  | Reference |
| Men | 0.69 (0.58, 0.82) | 2.22 (1.45, 3.41) | 1.16 (0.93, 1.44) |  | 3.22 (2.05, 5.05) | 1.68 (1.30, 2.17) |  | 0.52 (0.33, 0.83) |
| Residence |  |  |  |  |  |  |  |  |
| Urban | Reference | Reference | Reference |  | Reference | Reference |  | Reference |
| Rural | 1.11 (0.99, 1.24) | 0.73 (0.54, 1.00) | 1.06 (0.91, 1.23) |  | 0.66 (0.48, 0.91) | 0.95 (0.80, 1.13) |  | 1.44 (1.03, 2.02) |
| Education |  |  |  |  |  |  |  |  |
| College or university | Reference | Reference | Reference |  | Reference | Reference |  | Reference |
| Middle or high school | 1.29 (0.94, 1.77) | 0.99 (0.49, 2.02) | 1.36 (0.91, 2.03) |  | 0.77 (0.36, 1.64) | 1.05 (0.65, 1.69) |  | 1.37 (0.62, 3.03) |
| Primary school or below | 1.34 (0.97, 1.86) | 1.12 (0.53, 2.36) | 1.34 (0.88, 2.05) |  | 0.83 (0.37, 1.85) | 1.00 (0.61, 1.65) |  | 1.20 (0.52, 2.79) |
| Smoking |  |  |  |  |  |  |  |  |
| Never | Reference | Reference | Reference |  | Reference | Reference |  | Reference |
| Former | 0.88 (0.67, 1.15) | 0.70 (0.39, 1.26) | 0.83 (0.60, 1.14) |  | 0.80 (0.42, 1.49) | 0.95 (0.64, 1.39) |  | 1.19 (0.62, 2.27) |
| Current, cigarettes (or equivalent)/d | |  |  |  |  |  |  |  |
| <20 | 0.87 (0.71, 1.08) | 0.66 (0.39, 1.09) | 0.66 (0.50, 0.86) |  | 0.75 (0.44, 1.29) | 0.75 (0.55, 1.03) |  | 1.00 (0.57, 1.76) |
| ≥20 | 0.93 (0.76, 1.15) | 0.66 (0.40, 1.08) | 0.81 (0.63, 1.04) |  | 0.70 (0.42, 1.19) | 0.87 (0.64, 1.18) |  | 1.24 (0.72, 2.12) |
| Alcohol drinking |  |  |  |  |  |  |  |  |
| Less than weekly | Reference | Reference | Reference |  | Reference | Reference |  | Reference |
| Former | 1.20 (0.91, 1.58) | 1.17 (0.61, 2.25) | 1.82 (1.33, 2.48) |  | 0.97 (0.49, 1.94) | 1.51 (1.04, 2.21) |  | 1.56 (0.77, 3.15) |
| Weekly | 1.29 (1.03, 1.61) | 1.13 (0.61, 2.07) | 1.50 (1.15, 1.95) |  | 0.87 (0.46, 1.65) | 1.16 (0.84, 1.60) |  | 1.33 (0.69, 2.55) |
| Daily, g/d (pure alcohol) |  |  |  |  |  |  |  |  |
| <30 | 0.96 (0.67, 1.40) | 1.72 (0.87, 3.42) | 1.28 (0.83, 1.99) |  | 1.78 (0.84, 3.79) | 1.33 (0.78, 2.27) |  | 0.74 (0.34, 1.64) |
| 30–59 | 1.13 (0.82, 1.56) | 1.08 (0.51, 2.31) | 1.45 (0.99, 2.12) |  | 0.96 (0.43, 2.14) | 1.28 (0.80, 2.04) |  | 1.34 (0.58, 3.07) |
| ≥60 | 1.50 (1.14, 1.97) | 1.31 (0.65, 2.64) | 1.57 (1.11, 2.23) |  | 0.87 (0.42, 1.82) | 1.05 (0.70, 1.58) |  | 1.21 (0.56, 2.60) |
| Fresh vegetable consumption |  |  |  |  |  |  |  |  |
| Daily | Reference | Reference | Reference |  | Reference | Reference |  | Reference |
| Nondaily | 1.18 (0.94, 1.49) | 1.25 (0.65, 2.40) | 1.17 (0.87, 1.58) |  | 1.05 (0.53, 2.08) | 0.99 (0.70, 1.41) |  | 0.94 (0.47, 1.91) |
| Fresh fruit consumption |  |  |  |  |  |  |  |  |
| Daily | Reference | Reference | Reference |  | Reference | Reference |  | Reference |
| Nondaily | 1.20 (1.03, 1.41) | 1.48 (0.95, 2.29) | 1.25 (1.01, 1.54) |  | 1.22 (0.78, 1.93) | 1.04 (0.81, 1.32) |  | 0.85 (0.53, 1.36) |
| Red meat consumption |  |  |  |  |  |  |  |  |
| Daily | Reference | Reference | Reference |  | Reference | Reference |  | Reference |
| Weekly | 1.01 (0.89, 1.15) | 1.03 (0.73, 1.46) | 1.01 (0.85, 1.20) |  | 1.02 (0.71, 1.47) | 1.00 (0.82, 1.22) |  | 0.98 (0.67, 1.44) |
| Less than weekly | 1.10 (0.92, 1.31) | 0.84 (0.49, 1.45) | 1.20 (0.95, 1.51) |  | 0.77 (0.44, 1.35) | 1.10 (0.84, 1.43) |  | 1.43 (0.80, 2.56) |
| Total physical activity^*^ |  |  |  |  |  |  |  |  |
| Low | Reference | Reference | Reference |  | Reference | Reference |  | Reference |
| Medium | 1.06 (0.94, 1.19) | 1.04 (0.74, 1.46) | 0.86 (0.73, 1.00) |  | 0.98 (0.69, 1.39) | 0.81 (0.68, 0.97) |  | 0.82 (0.57, 1.18) |
| High | 1.04 (0.92, 1.18) | 1.01 (0.70, 1.47) | 0.83 (0.70, 0.98) |  | 0.97 (0.66, 1.43) | 0.80 (0.66, 0.97) |  | 0.82 (0.55, 1.22) |
| BMI |  |  |  |  |  |  |  |  |
| <18.5 kg/m^2^ | Reference | Reference | Reference |  | Reference | Reference |  | Reference |
| 18.5–23.9 kg/m^2^ | 2.40 (1.67, 3.44) | 1.10 (0.50, 2.41) | 2.54 (1.45, 4.46) |  | 0.46 (0.20, 1.08) | 1.06 (0.55, 2.04) |  | 2.31 (0.89, 6.01) |
| 24.0–27.9 kg/m^2^ | 4.41 (3.07, 6.34) | 2.14 (0.97, 4.73) | 6.18 (3.53, 10.84) |  | 0.49 (0.21, 1.15) | 1.40 (0.73, 2.69) |  | 2.88 (1.10, 7.53) |
| ≥28.0 kg/m^2^ | 6.75 (4.63, 9.84) | 2.84 (1.21, 6.66) | 10.64 (5.99, 18.90) |  | 0.42 (0.17, 1.05) | 1.58 (0.81, 3.08) |  | 3.75 (1.36, 10.35) |
| WC (men/women) |  |  |  |  |  |  |  |  |
| <85/80 cm | Reference | Reference | Reference |  | Reference | Reference |  | Reference |
| 85–90/80–85 cm | 1.75 (1.56, 1.97) | 1.86 (1.32, 2.62) | 2.09 (1.78, 2.45) |  | 1.06 (0.74, 1.52) | 1.19 (0.99, 1.44) |  | 1.13 (0.78, 1.63) |
| ≥90/85 cm | 2.01 (1.80, 2.26) | 1.88 (1.33, 2.64) | 2.98 (2.57, 3.44) |  | 0.93 (0.66, 1.33) | 1.48 (1.25, 1.75) |  | 1.58 (1.10, 2.28) |
| Body shape |  |  |  |  |  |  |  |  |
| BMI<18.5 | 0.42 (0.29, 0.60) | 0.90 (0.41, 1.98) | 0.40 (0.23, 0.71) |  | 2.16 (0.92, 5.07) | 0.97 (0.50, 1.86) |  | 0.45 (0.17, 1.17) |
| BMI 18.5–23.9, WC<90/85 | Reference | Reference | Reference |  | Reference | Reference |  | Reference |
| BMI 18.5–23.9, WC≥90/85 | 1.08 (0.66, 1.75) | 0.60 (0.08, 4.39) | 2.41 (1.42, 4.07) |  | 0.56 (0.07, 4.25) | 2.24 (1.15, 4.35) |  | 4.00 (0.52, 30.76) |
| BMI 24.0–27.9, WC<90/85 | 1.85 (1.65, 2.07) | 1.96 (1.40, 2.73) | 2.37 (2.04, 2.77) |  | 1.06 (0.75, 1.50) | 1.29 (1.08, 1.54) |  | 1.21 (0.85, 1.74) |
| BMI 24.0–27.9, WC≥90/85 | 1.85 (1.59, 2.16) | 1.88 (1.21, 2.91) | 2.85 (2.35, 3.45) |  | 1.01 (0.65, 1.59) | 1.54 (1.23, 1.93) |  | 1.51 (0.95, 2.42) |
| BMI≥28.0, WC<90/85 | 2.67 (1.91, 3.75) | 2.48 (0.88, 6.98) | 3.31 (2.12, 5.17) |  | 0.93 (0.32, 2.68) | 1.24 (0.75, 2.04) |  | 1.34 (0.44, 4.02) |
| BMI≥28.0, WC≥90/85 | 2.84 (2.44, 3.31) | 2.57 (1.62, 4.06) | 4.45 (3.67, 5.39) |  | 0.90 (0.56, 1.45) | 1.57 (1.26, 1.95) |  | 1.73 (1.07, 2.82) |

The table presents the adjusted RRR (95% CI). Please refer to the method section for detailed covariate adjustment.

^*^Total physical activity level was categorized based on age- (<50, 50–59, and ≥60 years) and sex-specific tertile cutoff points.

BMI, body mass index; DBP, diastolic blood pressure; RRR, relative risk ratio; SBP, systolic blood pressure; WC, waist circumference.
